# Semidiurnal Temperature Changes Caused by Tidal Front Movements in the Warm Season in Seabed Habitats on the Georges Bank Northern Margin and Their Ecological Implications

**DOI:** 10.1371/journal.pone.0055273

**Published:** 2013-02-06

**Authors:** Vincent G. Guida, Page C. Valentine, Leslie B. Gallea

**Affiliations:** 1 Northeast Fisheries Science Center J. J. Howard Laboratory, National Oceanic and Atmospheric Administration, National Marine Fisheries Service, Highlands, New Jersey, United States of America; 2 Woods Hole Coastal and Marine Science Center, United States Geological Survey, Woods Hole, Massachusetts, United States of America; National Institute of Water & Atmospheric Research, New Zealand

## Abstract

Georges Bank is a large, shallow feature separating the Gulf of Maine from the Atlantic Ocean. Previous studies demonstrated a strong tidal-mixing front during the warm season on the northern bank margin between thermally stratified water in the Gulf of Maine and mixed water on the bank. Tides transport warm water off the bank during flood tide and cool gulf water onto the bank during ebb tide. During 10 days in August 2009, we mapped frontal temperatures in five study areas along ∼100 km of the bank margin. The seabed “frontal zone”, where temperature changed with frontal movment, experienced semidiurnal temperature maxima and minima. The tidal excursion of the frontal boundary between stratified and mixed water ranged 6 to 10 km. This “frontal boundary zone” was narrower than the frontal zone. Along transects perpendicular to the bank margin, seabed temperature change at individual sites ranged from 7.0°C in the frontal zone to 0.0°C in mixed bank water. At time series in frontal zone stations, changes during tidal cycles ranged from 1.2 to 6.1°C. The greatest rate of change (−2.48°C hr^−1^) occurred at mid-ebb. Geographic plots of seabed temperature change allowed the mapping of up to 8 subareas in each study area. The magnitude of temperature change in a subarea depended on its location in the frontal zone. Frontal movement had the greatest effect on seabed temperature in the 40 to 80 m depth interval. Subareas experiencing maximum temperature change in the frontal zone were not in the frontal boundary zone, but rather several km gulfward (off-bank) of the frontal boundary zone. These results provide a new ecological framework for examining the effect of tidally-driven temperature variability on the distribution, food resources, and reproductive success of benthic invertebrate and demersal fish species living in tidal front habitats.

## Introduction

**Figure 1:**
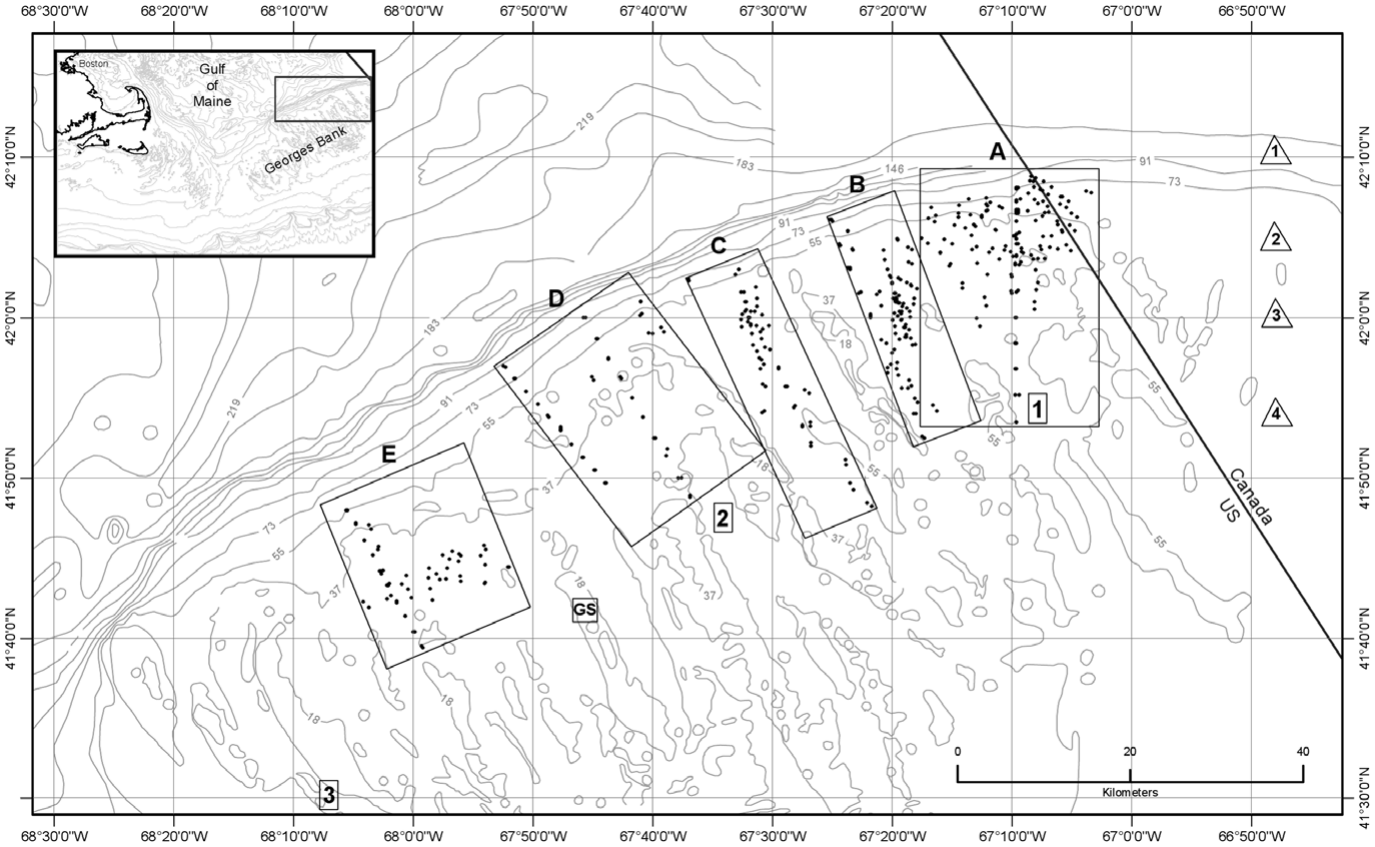
Map showing the location of Georges Bank (inset) and study areas. The locations of study areas A-E (boxes) and 464 seabed temperature stations (dots) occupied in the August 4–13, 2009 time period are indicated. Numbers 1–3 in rectangles mark tidal current prediction sites shown on NOAA Chart 13200 [43]. Numbers 1–4 in triangles mark locations of moored instruments of Loder et al. [18] in July 1988. GS marks location of tidal height predictions on Georges Shoal [42]. Isobaths from NOAA Chart 13200 [43] are labeled in meters.

Georges Bank is a large, relatively shallow offshore continental shelf feature that extends eastward from the southeastern Massachusetts coast toward the southern tip of Nova Scotia and forms the southern boundary of the much deeper Gulf of Maine (Fig. 1). It is one of the world’s most productive marine ecosystems [1]. The character and function of Georges Bank benthic habitats are influenced by spatial factors of hydrographic, geologic, climatic, and anthropogenic origin [2]–[4], producing a patchwork of distinct habitats that contribute to overall benthic, and ultimately, fisheries production [5].

The hydrographic setting for the benthic habitats of Georges Bank results from the interaction of water masses, tides, topography, and atmospheric warming and cooling. In the warm season, shallow tidally-mixed Georges Bank Water (GBW) is bounded on the north by a 3-layer system of water masses in the Gulf of Maine that includes Maine Surface Water (MSW), Maine Intermediate Water (MIW), and Maine Bottom Water (MBW); in the cool season, MSW and MIW coalesce and form the upper layer of a 2-layer system with MBW [6]. A clockwise gyre, driven by strong semidiurnal tidal currents, and influenced by wind and neighboring water masses, surrounds well-mixed water on the top of the bank [7], [8]. High primary productivity on the bank results when nutrient-rich water from the Gulf of Maine is transported up the steep northern slope of the bank and pumped into the euphotic zone on the bank’s northern margin [9]–[11] where it enters the gyre.

Hydrographic fronts, as detected from surface temperature gradients between water masses [visible in satellite imagery, are pervasive features of northwestern Atlantic continental margins [12], [13]. The front located along the northern margin of Georges Bank is typical of a class of tidal-mixing fronts that form at the edges of shallow banks. Turbulence generated by tidal currents at the seabed on the bank causes mixing of the water column, enabling a front to develop between mixed and stratified water masses and guaranteeing the front impinges directly on the seabed [14]–[16].

The boundary between the Georges Bank and Gulf of Maine water masses is a complex frontal system, best developed in the warm season, that is continually in motion within a narrow zone along the northern topographic edge of the bank in response to semidiurnal tidal forcing [17]–[19]. During flood tide (into the Gulf of Maine), the front moves northward in an off-bank direction, and during ebb tide, it moves southward in an on-bank direction. The semidiurnal tide causes 2 high and 2 low tides per tidal day of 24.83 hr [20], which result in marked changes in seabed water temperature four times a day in the study region. In this study, the term “frontal zone” describes the area of the seabed where the water temperature is changed by tidal movement of the front. The term “frontal boundary zone” describes the area swept by tidal movement of the transitional boundary between stratified and mixed water. The area of the frontal zone is larger than that of the frontal boundary zone. The front is particularly well developed during the summer and early fall when temperature gradients are largest between warm, well-mixed Georges Bank Water and cold, stratified Maine Intermediate Water [18], and when the bank’s gyre circulation is most rapid [21]. The intensity, persistence, and extent of this summer front are evident in satellite thermal imagery [12].

There is a substantial history of effort to understand circulation over the entirety of Georges Bank through hydrographic modeling [22], [23]. The most recently published *in situ* measurements and analyses of tidal front temperature phenomena on the northern margin of the bank have been made in Canadian waters [18], [19], [24], [25], where the bathymetry is somewhat deeper than in U.S. waters (see Figure 1 for locations of study sites of Loder et al., [18]). On the shallower and larger U.S. portion of the bank margin, published hydrographic data that describe the front’s temperature structure apparently are limited to one time-series station from October 1978 [26].

Tidal hydrology and circulation patterns influence the ecology of the bank and play a role in nutrient supply and primary production [27]–[29] and in the distribution of holozooplankton and larvae [26], [30]–[34]. In the area of the northern bank margin affected by the movement of the front, seabed temperature at an individual seabed site was reported to change 6 to 7°C from high to low tide and temperature rates of change were as large as 4°C hr^−1^ [26]. The influence of rapid cyclic temperature changes on benthic communities of the bank and on the demersal fishes they support is unknown.

Several tidal fronts located in the coastal seas of northern Europe are similar to the northern Georges Bank front and have been studied extensively [35], [36]. These fronts have been shown to coincide with changes in the distributions of bivalve species [37], epibenthic invertebrates and fish [38], and sediment texture and sedimentary processes [39]. Elements of the benthic and demersal faunas have been shown to be enriched in the immediate vicinity of the fronts [38], [40]. However, the tidal-mixing fronts in northern Europe have been treated as quasi-fixed features, and there has been little documentation of their semidiurnal tidal motions [41] or of the biological consequences of such movements. In contrast, the semidiurnal dynamism of the Georges Bank front has been a focus of previous studies but restricted to a small area only ([18]: Fig. 1). Here we expand on those studies to document frontal movement and attendant temperature changes along ∼100 km of the bank’s northern margin habitats.

The purposes of the present study are to quantify and map temperature changes at the seabed in U.S. waters on the northern margin of Georges Bank caused by movement of a strong semidiurnal tidal front in the warm season; to discuss the potential for applying these results to the study and management of the productive fisheries species and habitats located there; and to provide *in situ* temperature data that can be used to calibrate models of hydrodynamic processes in the frontal zone.

## Methods

### 1. Ethics Statement

The field work reported herein and the use of ship time to perform it were authorized by Dr. Nancy B. Thompson, Science and Research Director of the National Oceanic and Atmospheric Administration (NOAA), National Marine Fisheries Service, Northeast Fisheries Science Center (NEFSC) and Captain Emily Christman, Commanding Officer, NOAA Marine Operations Center-Atlantic through written approval of a detailed cruise plan prior to departure. The work was also undertaken with the knowledge of Dr. William Schwab, Chief Scientist, U.S. Geological Survey Woods Hole Science Center. All sampling was performed within the U.S. and Canadian Exclusive Economic Zones (EEZs), but outside the waters of all States, Provinces and marine sanctuaries. Entry into and sampling within the Canadian EEZ was authorized specifically for this cruise by the government of Canada via an application process through the U.S. Department of State. Shipboard operations were conducted in accordance with NOAA and NEFSC protocols, which incorporate the U.S. Government’s Code of Environmental Management Principles (CEMP) for pollution prevention. Protocols to limit impacts from sampling, ship’s acoustic emissions, and unintentional encounters with protected species were reported to and reviewed by U.S. Environmental Protection Agency (EPA) under an Environmental Assessment (EA) process for the larger NEFSC shipboard sampling program. The work reported upon here involved no intentional take of organisms and minimal disturbance of physical habitats. All raw hydrographic data collected during in the course of this work is available at this address: http://www.nefsc.noaa.gov/epd/ocean/MainPage/ioos.html. To access the data, select “Water Column Properties”/”By Cruise ID”/”DEL0908-05-AUG-2009”.

### 2. Study Sites

All field data presented here were collected from August 4 to 13, 2009 during cruise DE09-08 aboard NOAA Fisheries Vessel *Delaware II*. Sites were located along the northern margin of the U.S. sector of Georges Bank in areas where studies of fisheries habitats are in progress (Fig. 1). Substrates in the study region are dominated by sand and gravel, with extensive gravel pavements separated by sand ridges which are oriented northwest-southeast, parallel to the strongest tidal flow. At present, habitat research is focused in study areas A, B, and C in the east, and more temperature data were collected there than in areas D and E where study is just commencing. Hydrographic transects were positioned in each study area so as to lie parallel to the movement (on-bank and off-bank) of the major semidiurnal tidal current and transverse to the orientation (along-bank) of the associated semidiurnal tidal front. Sites of additional temperature observations were selected based on the need to image and sample the seabed for habitat studies. Sample sites lie in water depths of 27 to 94 m, and the 5 study areas are located along approximately 100 km of the bank margin which trends northeast-southwest in the study region. Ship and station positions for all data collected on the cruise were recorded using Global Positioning System (GPS) with an accuracy of approximately 12 m.

Meteorological observations taken during the cruise indicated that weather was not a major influence on hydrographic conditions. No storms occurred on Georges Bank, and based on 10-minute averages of true wind velocities, mean wind velocity was 4.77 ms^−1^ (SD = 1.92 ms^−1^), with a maximum value of 10.06 ms^−1^ (moderate breeze on the Beaufort scale). Wind direction showed no dominant heading. Tidal height predictions for Georges Shoal ([42]: Fig. 1) suggested relatively uniform tidal forcing with an amplitude of approximately 1.2 (±0.1) m over the period of this study. That period (August 4–13, 2009) missed spring and neap variations in tidal amplitudes (up to ±0.5 m) predicted for later in the month.

### 3. Choice of Biologically Relevant Data

Our goal has been to identify patterns of ecological data that are relevant to the management of demersal fisheries on Georges Bank. The entire range of salinity measured via vertical CTD casts in our study areas was 31.66 to 33.06 psu (n = 22,356 measurements). The small variations within these limits are not likely to have appreciable influence on distribution and behavior of demersal species. By contrast, seabed temperature values within the same dataset ranged from 5.1° to 16.4°C, a very large span in physiological terms. We have chosen to evaluate patterns in the distribution and dynamics of seabed temperature exclusively in the current work. Temperature data recorded at sites along near-synoptic CTD transects across the tidal front and at time-series stations in the frontal zone are sufficient for mapping the broad areal patterns of tidally-influenced seabed water temperature changes in which we are interested. They do not allow the mapping of the very high-frequency temperature fluctuations known to characterize the water column of the frontal system [18].

### 4. The Timing of High and Low Tides in the Study Areas

Investigation of the internal temperature structure and movement of the tidal front on the northern margin of Georges Bank requires knowledge of the timing of high and low tides in the study areas in order to determine when to sample the water column. At three locations in the study region, NOAA Chart 13200 displays a diagram showing 12 vectors that represent the direction and velocity of tidal currents at hourly intervals ([43], Fig. 1). The predicted times of high and low tides (slack water) are represented by the shortest tidal current velocity vectors on these diagrams. The actual time of each hourly vector shown in the diagrams is related to the time of maximum flood tide at Pollock Rip Channel (east end) near Cape Cod [44]. For example, on a given day at location 1 (Fig. 1), high tide and low tide occur 5.0 and 11.0 hours, respectively, after the time of maximum flood tide at Pollock Rip Channel. At location 2, the equivalent high and low tide times relative to Pollock Rip Channel are 4.5 and 10.5 hours and at location 3, 4.0 and 10.0 hours. This information was used to construct tables of high and low tide times for use in the field and later for determining the time before or after high tide of each temperature observation.

### 5. Vertical Temperature Profiles Collected Along Transects Across the Tidal Front

Water column properties were collected across the front in 5 study areas on the northern margin of the bank (Fig. 1) using a Seabird Electronics® SBE Model 19+ profiling CTD deployed vertically from the ship by a conducting hydrographic wire. The instrument was lowered to approximately 1 to 5 m above the seabed and retrieved at a rate of approximately 0.8 m s^−1^ second while recording the water conductivity, temperature, and depth (CTD). Data were collected at a rate of 2 observations per second during retrieval (upcast) of the profiler. The sensors in the profiler recorded temperature to an accuracy of 0.0001°C and depth to an accuracy of 0.001 m. The maximum water depth at a station was recorded by the ship’s Simrad® EK60-120 kHz echo sounder to an accuracy of 0.01 m.

**Figure 2:**
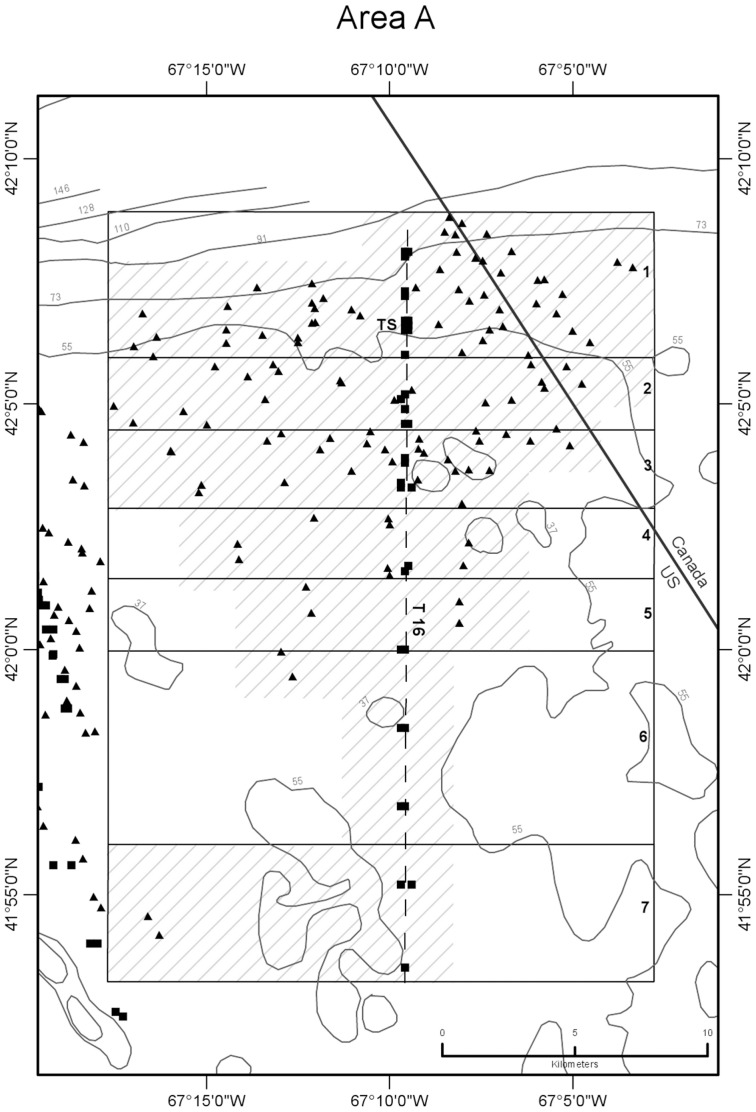
Detail map of study area A showing subareas and seabed temperature stations. Data was obtained from the northern margin of Georges Bank in August 2009. Subareas 1–7 of are shown as boxes subdividing the larger area A box, labeled just inside their right (eastern) margin. Dashed line is path of CTD transect 16 that was sampled around high and low tides (Fig. 7A, Table 1). TS is the site of time-series station (Fig. 8, Tables 3, 4). Hachures show distribution of temperature data used to characterize the effect of frontal movement in each subarea (Fig. 11, Table 5). Black squares are hydrocast temperature observations on transects and at time-series stations. Black triangles are temperature observations at the starts and ends of video drift stations. Isobaths from NOAA Chart 13200 [43] are labeled in meters.

**Figure 3:**
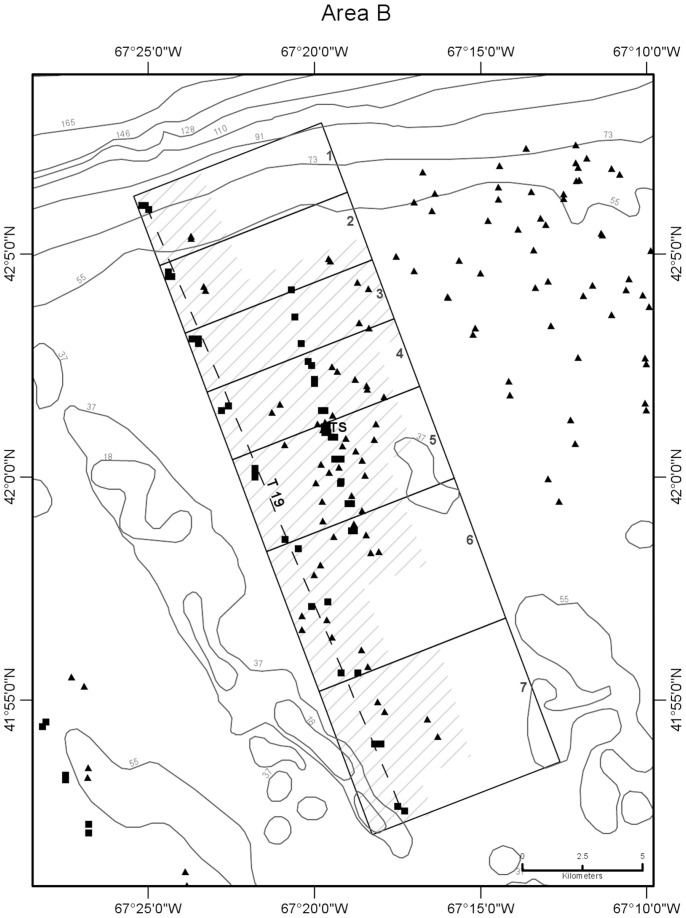
Detail map of study area B showing subareas and seabed temperature stations. Data were obtained from the northern margin of Georges Bank in August 2009. Subareas 1–7 are shown as boxes subdividing the larger area B box, labeled just inside their right (northeastern) margin. Dashed line is path of CTD transect 19 that was sampled around high and low tides (Fig. 7B, Table 1). TS is the site of time-series station (Fig. 8, Table 3). Other symbols and depth contours are as in Fig. 2.

**Figure 4:**
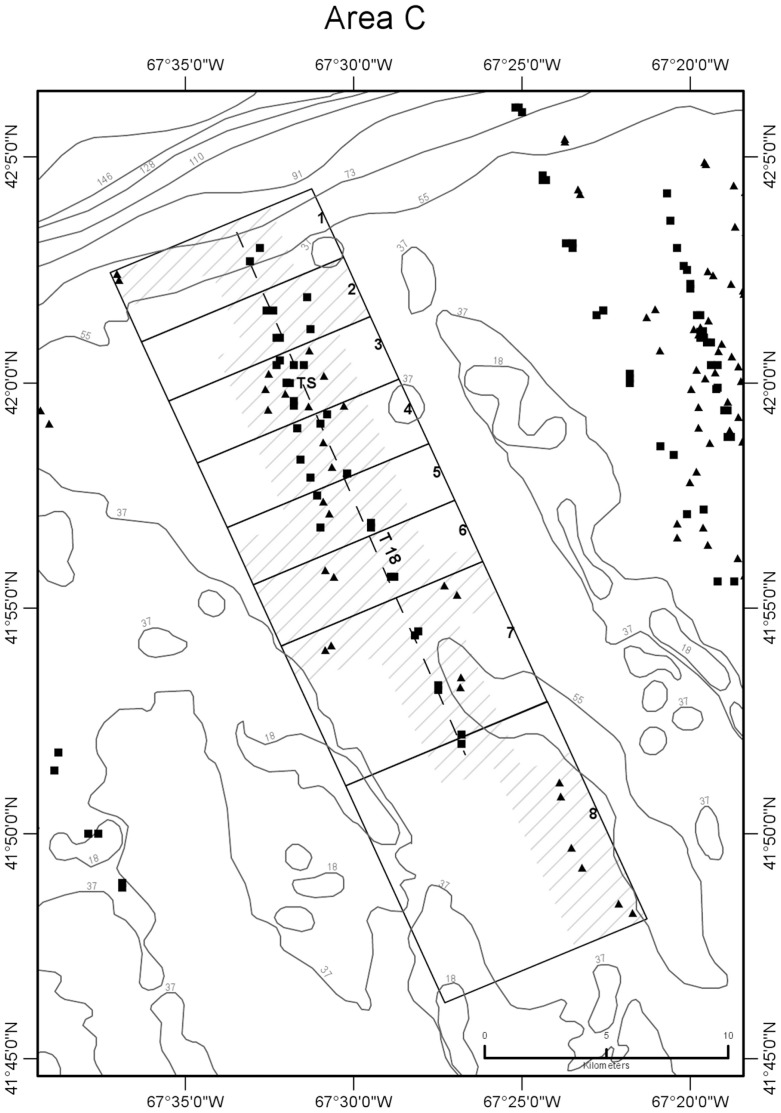
Detail map of study area C showing subareas and seabed temperature stations. Data were obtained from the northern margin of Georges Bank in August 2009. Subareas 1–8 of are shown as boxes subdividing the larger area C box, labeled just inside their right (northeastern) margin. Dashed line is path of CTD transect 18 that was sampled around high and low tides (Fig. 7C, Table 1). TS is the site of time-series station (Fig. 8, Table 3). Other symbols and depth contours are as in Fig. 2.

**Figure 5:**
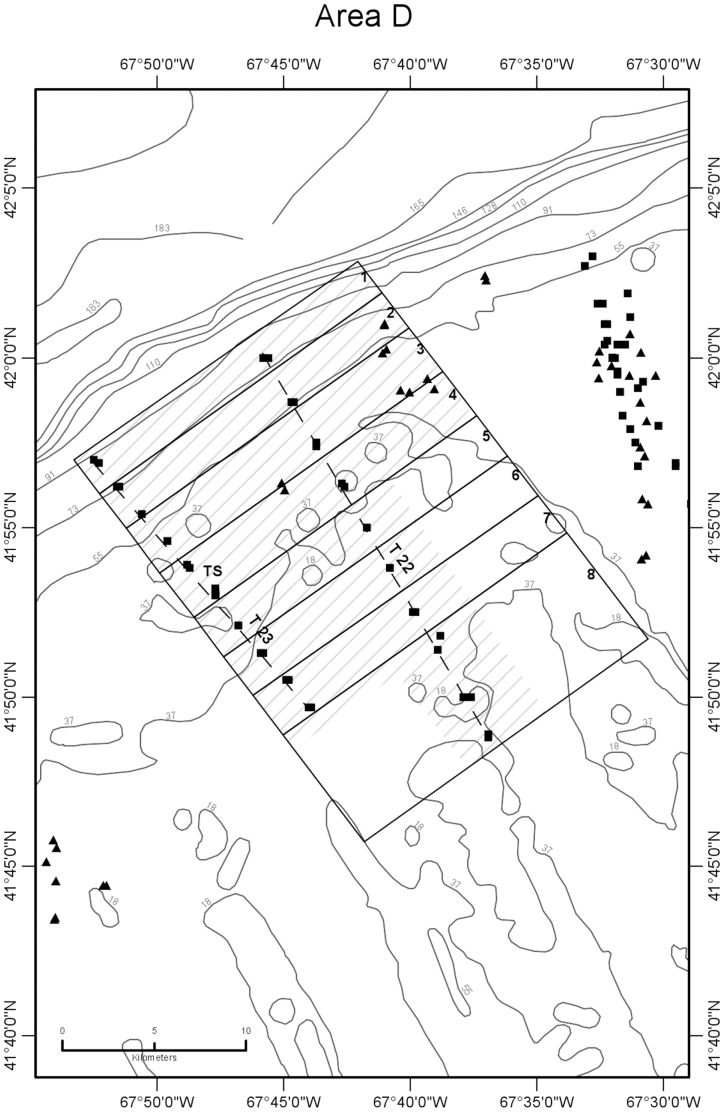
Detail map of study area D showing subareas and seabed temperature stations. Data were obtained from the northern margin of Georges Bank in August 2009. Subareas 1–8 are shown as boxes subdividing the larger area D box, labeled just inside their right (northeastern) margin. Dashed lines are paths of CTD transects 22 and 23 that were sampled around high and low tides (Figs. 7b D and E, Table 1). TS is the site of time-series station (Fig. 8, Table 3). Other symbols and depth contours are as in Fig. 2.

**Figure 6:**
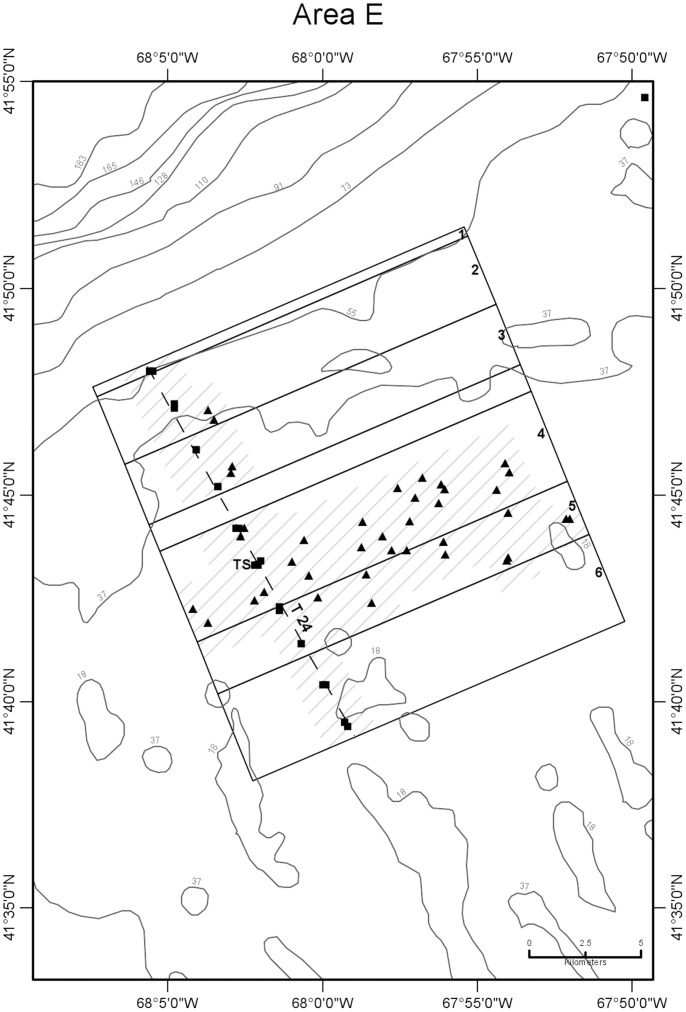
Detail map of study area E showing subareas and seabed temperature stations. Data were obtained from the northern margin of Georges Bank in August 2009. Subareas 1–6 are shown as boxes subdividing the larger area E box, labeled just inside their right (northeastern) margin. Dashed line is the path of CTD transect 24 that was sampled around high and low tides (Fig. 7F, Table 1). TS is the site of time-series station (Fig. 8, Table 3). Other symbols and depth contours are as in Fig. 2. Data was insufficient to characterize the un-numbered subarea between subareas 3 and 4.

**Figure 7:**
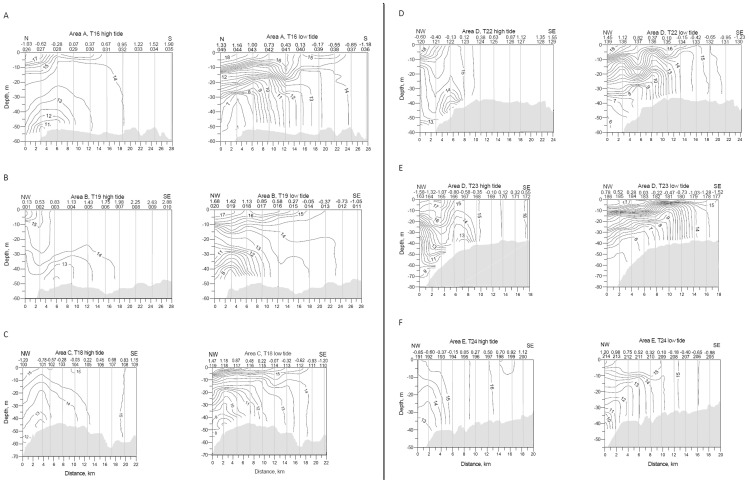
Water-column cross sections along CTD transects in study areas A-F. Data were obtained from the northern margin of Georges Bank in August 2009 (Figs. 2,3,4,5,6). Stations were occupied in numerical order, from off-bank to on-bank around high tide and from on-bank to off-bank around low tide. Temperature contour interval is 0.5°C. All sections are oriented from N or NW (left, off bank) toward S or SE (right, on bank). Numbered vertical lines represent CTD temperature profile stations. Depth of deepest station is not shown when results of contouring were unreliable due to a large change in depth of seabed between some off-bank stations. See Table S1 for station depths. Numbers above station numbers indicate times of temperature observations in hours before (-) and after (+) high tide or low tide, depending on which tide is represented.

**Figure 8:**
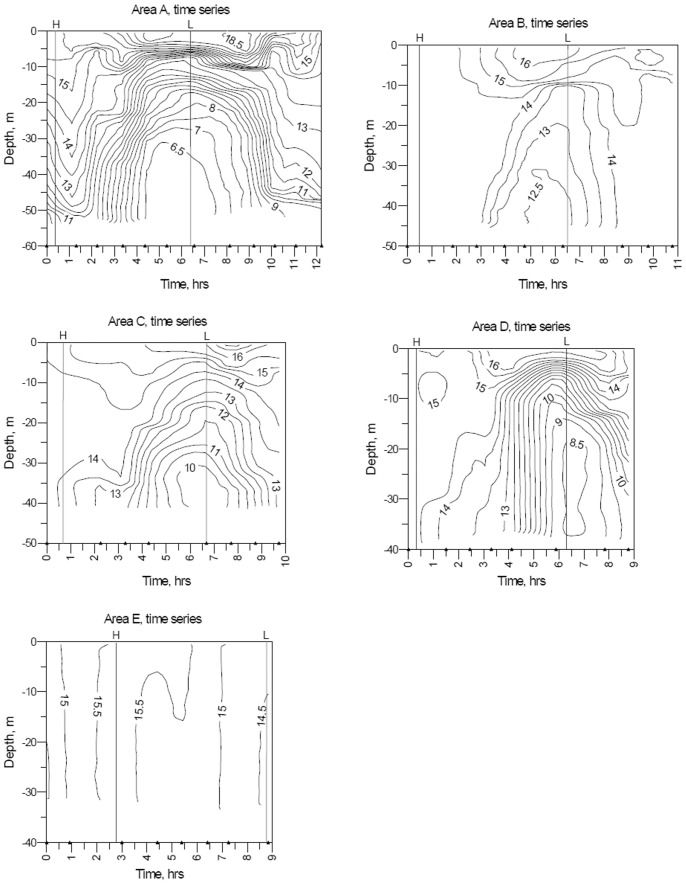
Water-column cross sections at CTD time-series sites in study areas A-E. Data were obtained from the northern margin of Georges Bank in August 2009 (Figs. 2,3,4,5,6). Observations were made over time periods of 8.8 to 12. 2 hr (Tables 3, 4). Temperature contour interval is 0.5°C. Vertical lines represent time of high (H) and low (L) tide. Black triangles on the x-axis indicate times of temperature observations after start of time series.

CTD profile stations were located along transects oriented normal (north-south or northwest-southeast) to the trend of the shelf edge so as to obtain data for constructing temperature sections across the front (Figs. 2,3,4,5,6). Individual casts were made at spatial intervals ranging from 2 to 3 km, depending upon transect length, and timed around predicted times of local high and low tides (Fig. 7). Along each transect, two casts (paired stations) were completed at each site during a 12-hour period to document the frontal temperature structure around the predicted times of high and low tides. Additionally, in each of the 5 study areas, during the interval between flood and ebb tide passes, a time series of CTD casts was performed on an hourly or half-hourly basis (Fig. 8) at a station located on or near transects T16, T18, T19, T23, and T24 to record the temperature structure of the front as it moved past the location (Figs. 2,3,4,5,6).

CTD stations on a transect could not be performed simultaneously at the time of the predicted high or low tide. Transects were completed as rapidly as possible, and with the exception of 1 station on transect 16 in study area A and 5 stations on transect 19 in study area B, all CTD casts associated with flood and ebb tide passes were made within 100 minutes of the predicted times of slack tidal currents. Flood tide passes along transects were begun at their northernmost (off-bank) ends, starting 90 min prior to predicted slack high water. Sampling proceeded southward from there as the front completed its northward (off-bank) movement. Ebb tide passes were begun at the southernmost (on-bank) ends of transects, starting 90 min prior to predicted slack low water, and sampling proceeded northward as the front completed its southward (on-bank) movement. Six transects that included both a flood and ebb tide pass were completed in study areas A (T16), B (T19), C (T18), D (T22, T23), and E (T24). In all, 216 CTD stations were completed in the 5 study areas (A-E) along the northern margin of the bank in water depths ranging from 32 to 94 m during a 9-day period from August 5 to 13, 2009.

### 6. Analysis of the Temperature Structure and Movement of the Tidal Front

At each CTD station, temperature data were collected from the maximum depth of the instrument (1–5 m above the seabed) to the sea surface. After inspection, data from shallower than 1 m below the sea surface were considered to be unreliable and discarded. The temperature structure of the tidal front along each transect at approximately flood and ebb tides was mapped by using Surfer® software (Golden Software, Inc.) to contour temperature data collected at individual stations by water depth and distance between stations (Fig. 7A-F). Movement of the front during sampling along a transect caused unavoidable minor distortion in the contoured temperature data. The changing temperature structure of the front with time at a single location during ebb and flood tides was mapped by contouring temperature data collected multiple times from that location by water depth and time (Fig. 8). These temperature contour plots show water column structure (stratified or mixed), frontal movement on and off the bank, and variation in seabed temperature at individual locations in the frontal zone.

### 7. Temperature Data Collected on Seabed Video Transects

Seabed water temperature data were collected using a Seabird Electronics SBE Model 19 profiling CTD attached to the USGS’ Seaboss (seabed observation and sampling system [45], which was used to collect photographs and video imagery while drifting over the seabed in the study areas. The Seaboss and CTD were lowered from the ship to a depth approximately 1 m above the seabed, and water conductivity, temperature, and depth (CTD) data were collected during the entire track of each video drift, which ranged in length from 117 to 2664 m. Data were collected at a rate of 2 observations per second and were downloaded from the profiler after recovery. The sensors in the profiler aboard Seaboss were the same as those for the CTD used for vertical casts (Results section 4.). The maximum water depth at a station was recorded by the ship’s Simrad EK60-120 kHz echo sounder to an accuracy of 0.01 m.

**Figure 9:**
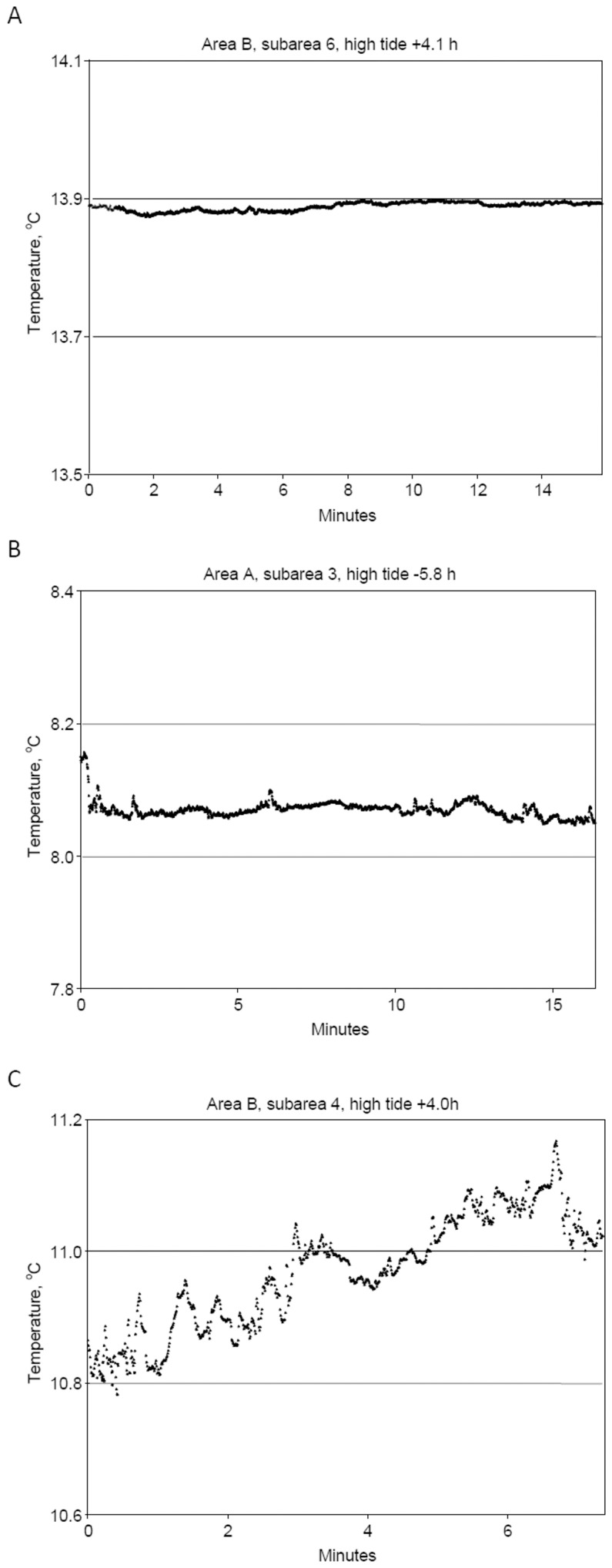
Hydrological complexity in CTD temperature data collected along Seaboss video drift tracks. A. Well-mixed, warm on-bank water showing little overall temperature change (<0.1°C); sta. 908007, 775 m drift. B. Within an hour of low tide in on- and off-bank areas, small high-frequency temperature changes (up to 0.02°C); sta. 908026, 259 m drift. C. Within 2 to 5 hours of high tide in off-bank areas, moderate high-frequency temperature changes (up to 0.1°C); sta. 908023, 304 m drift.

**Figure 10:**
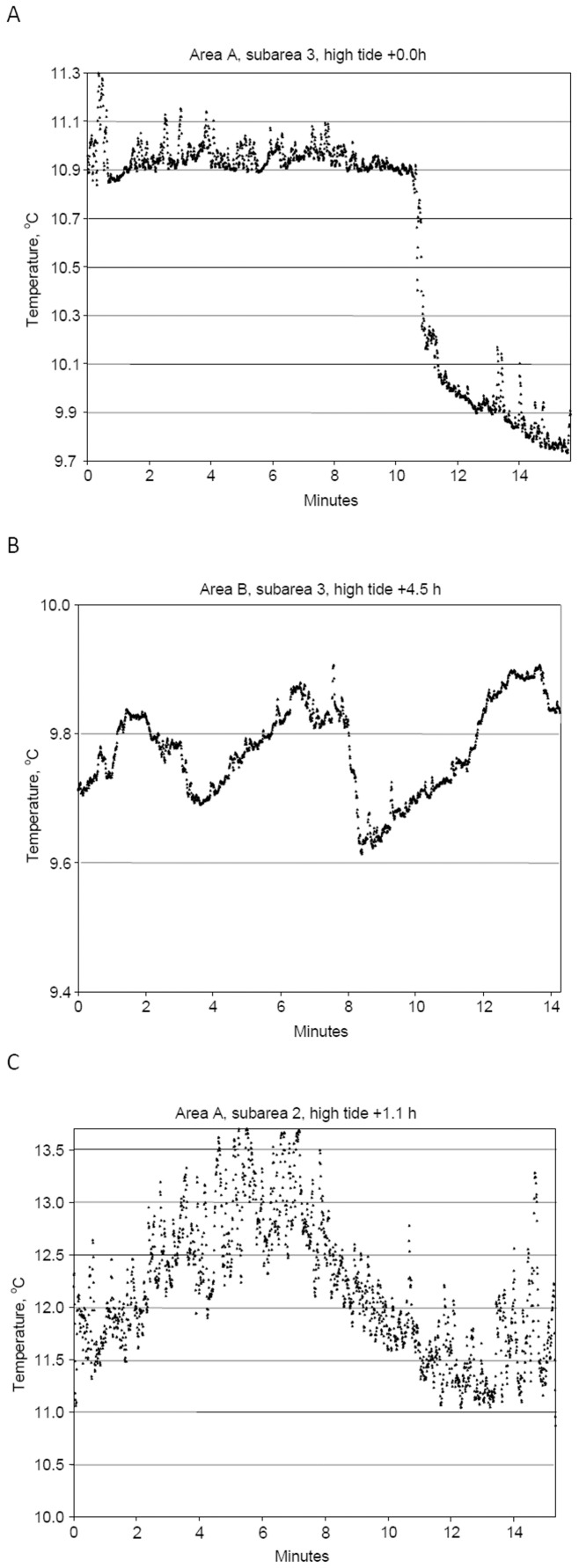
Rapid temperature changes in CTD temperature data collected along Seaboss video drift tracks. Examples of rapid temperature changes within the tidal front in off-bank subareas. A. Single rapid temperature changes (up to 0.6°C); sta. 908047, 801 m drift. B. Multiple rapid (4–5 min duration) saw tooth fluctuations (up to 0.3°C); sta. 908024, 498 m drift. C. Multiple rapid (6 min duration) saw tooth temperature fluctuations (up to 2–3°C); sta. 908034, 812 m drift.

Temperature observations were collected in five study areas (A-E) along the northern margin of the bank in water depths ranging from 27 to 87 m during a 10-day period from August 4 to 13, 2009 (Figs. 2,3,4,5,6). Seabed temperature observations (248 in all) collected at the end of the descent and the beginning of the ascent of the Seaboss supplemented those collected by 216 CTD casts. Temperature data collected at the seabed along Seaboss drift tracks were plotted by temperature and drift time to determine if high-frequency temperature changes were present that would suggest the presence of turbulent mixing in the frontal zone (Figs. 9, 10).

### 8. Analysis of Seabed Temperatures in Subareas of the Tidal Front Region

We hypothesized that the magnitude of temperature change at the seabed caused by tidally-driven frontal movement would vary with location in the frontal zone. For purposes of identifying this cross-frontal pattern, we initially divided each study area (A-E) into subareas of equal length (2.8 km) along the axis perpendicular to the front. An inspection of the distribution of our sampling points (Fig. 1), the temperatures observed at sites within each subarea, and subsequent plotting of these temperatures relative to their time of occurrence during flood and ebb tides (Fig. S1A-E), enabled us to identify and map geographic subareas within study areas A-E (Figs. 2,3,4,5,6) that experienced differing ranges of temperature change during movement of the front.

## Results

### 1. Water Column Temperature Structure of the Tidal Front at High and Low Tides

Contoured water column temperature data, collected at 2- to 3-km intervals along 6 transects across the northern bank edge, revealed the complex stratified structure of the tidal front (Fig. 7A-F). Stratification along transects was most pronounced at low tide when the front was at its maximum on-bank location. In the frontal zone, isotherms were primarily horizontal near the surface and more nearly vertical at depth. Near the off-bank end of most transects, upward-arching isotherms represented the advance and retreat of relatively cold stratified water from the Gulf of Maine onto the bank; and isolated patches of cold water were bounded on both sides by slightly warmer water at the seabed. The temperature of the water column varied as much as 12°C (6–18°C) from seabed to surface (Fig. 7A). At the on-bank ends of transects, the front was undetectable, the water column was well mixed, and widely-spaced near-vertical isotherms ranged from 14 to 16°C. The leading edge of the front, the frontal boundary, was represented by the location of near-vertical isotherms that separated stratified off-bank water from mixed on-bank water. Individual isotherms that intersected the seabed within the frontal boundary zone moved 6 (Fig. 7A, B) to 10 km (Fig. 7D) between high and low tides.

Some transect stations were located in the frontal zone, and others were located at all times in mixed bank water and were minimally affected by changes in temperature between tides (Fig. 7A-F). We differentiated seabed stations that were thermally affected by movement of the front from those that were not by comparing changes in temperature at paired stations at high and low tides along each transect. As we are interested in the potential effects of temperature and temperature variability on the biota, for the purpose of this study we classified those stations having a flood- vs. ebb-tide temperature difference of >1°C as being affected by the front (Table 1, S1). Other observers may select different criteria for classifying the stations, depending on their research goals. Using our definition, 5 of the 6 transects (T16, T18, T19, T22, T24) collected samples across the frontal boundary at high and low tides (Figs 7a A-C, 7b D, F). Transect T23 sampled only within the frontal zone, as the most bankward station pair (172 and 177) had a flood- vs. ebb-tide temperature change of 1.1°C (Figs. 5, 7b E, Table 1). The high- vs. low-tide temperature change at the most-affected pair of sites on each transect ranged from 3.7°C in area E to 7.0°C in area D (Table 1).

**Table 1:** Water depth, seabed temperature, and temperature change along transects across the tidal front.

Location	Stations		Seabed temperature °C			Frontal influence
Area, Transect	Pair	Depth	H Temp	L Temp	H-L ΔT	(Y/N)
	groups	range, m	Range	range		
A, T16	1–6	51–81	9.8–13.6	5.8–12.4	1.2–4.3	Y
	7–10	52–59	14.0–14.2	13.4–14.1	0.0–0.6	N
B, T19	1–3	51–82	9.5–13.2	6.1–9.5	2.9–6.1	Y
	4–10	47–57	12.8–14.3	12.3–14.2	0.1–0.7	N
C, T18	1–8	43–68	11.2–15.0	7.2–13.8	1.2–4.4	Y
	9–10	52–60	15.0	14.1–14.4	0.6–0.9	N
D, T22	1–7	37–88	11.2–15.0	7.2–13.8	1.2–4.4	Y
	8–10	37–45	15.7	15.1–15.7	0.0–0.6	N
D, T23	1–10	37–94	7.2–16.0	5.1–14.9	1.1–6.3	Y
E, T24	1–6	37–60	12.1–15.6	8.4–14.4	1.2–3.7	Y
	7–10	32–35	15.8–16.4	14.8–15.7	0.6–1.0	N

Temperature change from high to low tide is shown for the parts of transects where seabed temperature is influenced (Y) or not influenced (N) by frontal movement. Station pair groups (column 2) are numbered in each area from north to south (1–10; i.e. off-bank to on-bank). Each pair group contains up to 8 pairs of CTD stations, at which temperature was recorded at high or low tide. H Temp and L Temp are the ranges of seabed temperatures within the pair group measured at high and low tide, respectively. H-L ΔT is the range of temperature differences between high and low tides at stations within the pair group. This value is the criterion on which frontal influence is based. If the minimum value for H-L ΔT is >1.0°C, the pair group is considered influenced by frontal movement. Note that all station pairs in area D, Transect 23 were influenced. Area names (A-E) are as in Figure 1; names of transects (TXX) across the tidal front are as in Figures 2,3,4,5,6. See Figure 7 for locations of individual stations along CTD transects. Table S1 includes data for all 60 station pairs.

Seabed temperature change with distance (C km^−1^) along the parts of transects affected by the front in areas A-E averaged 0.22 to 0.50°C km^−1^ at high tide and 0.37 to 0.55°C km^−1^ at low tide (Tables 2, S2). Along parts of transects in mixed water not affected by the front, temperature change averaged 0.00 to 0.07°C km^−1^ at high tide and 0.07 to 0.12°C km^−1^ at low tide). The rate of temperature change (°C km^−1^) in each area was consistently greater at low tide than at high tide.

**Table 2:** Comparison of parts of seabed transects along which temperature is affected or not affected by the tidal front at high and low tide.

		Part of transect AFFECTED by front				Part of transect NOT AFFECTED by front			
Location		Temperature, °C				Temperature, °C			
Area, transect	Tide	Dist., km	Min/Max	ΔT	°C km^−1^	Dist., km	Min/Max	ΔT	°C km^−1^
A, T16	Hi	17.1	9.8/13.6	3.8	0.22	10.8	14.0/14.1	0.1	0.01
A, T16	Lo	17.1	5.8/12.4	6.6	0.39	10.8	13.4/14.1	0.7	0.07
B, T19	Hi	7.2	9.5/12.4	2.9	0.40	19.9	12.9/14.3	1.4	0.07
B, T19	Lo	7.7	6.1/9.5	3.4	0.44	19.6	12.3/14.2	1.9	0.10
C, T18	Hi	18.5	11.2/15.0	3.8	0.21	2.6	15.0/15.0	0.0	0.00
C, T18	Lo	18.0	7.2/13.8	6.6	0.37	3.5	14.1/14.4	0.3	0.09
D, T22	Hi	17.1	7.9/15.7	7.8	0.46	7.0	15.7/15.7	0.0	0.00
D, T22	Lo	17.2	5.8/14.6	8.8	0.51	6.8	15.1/15.7	0.6	0.09
D, T23	Hi	17.5	7.2/16.0	8.8	0.50	–	–	–	–
D, T23	Lo	17.8	5.1/14.9	9.8	0.55	–	–	–	–
E, T24	Hi	11.2	12.1/15.6	3.5	0.31	7.1	15.8/16.3	0.5	0.07
E, T24	Lo	10.9	8.4/14.4	6.0	0.55	7.3	14.8/15.7	0.9	0.12

Seabed temperature change per kilometer is given for parts of transects where individual sites are affected or not affected by the tidal front. By our definition, a site is affected by frontal movement if the temperature change between high and low tides is >1°C (Table S1, H-L ΔT). For example, in study area A at high tide, the part of transect T16 affected by the front extended over a distance of 17.1 km. Temperature changed from a minimum of 9.8°C (in the deep part) to a maximum of 13.6°C (in the shallow part) over that distance. Thus at high tide, temperature changed 3.8°C over 17.1 km and the rate of change was 0.22°C km^−1^. In area D, all stations along transect 23 were affected by frontal movement. See Figure 7 for locations of stations along CTD transects. Table S2 contains additional data.

### 2. Water Column Temperature Structure at Single Sites during Tidal Flow

Time series temperature data were recorded at 5 sites in study areas A-E, each located in the frontal zone on or close to a CTD transect that crossed the tidal front (Figs. 2,3,4,5,6). Water column temperature data collected periodically at each site were used to determine: a) the minimum and maximum seabed temperatures observed; b) the temperature change with time (°C hr^−1^) at that location; and c) the residence time on the bank of cold off-bank water. The 5 time series included observations collected over periods of 8.8 to 12.2 hr (Fig. 8, Table 3). The longest time series is in study area A, and it best represents the temperature effect caused by frontal movement over the seabed.

**Table 3:** Rates of seabed temperature change at time-series sites in study areas A-E.

	Date	Stations		Time-series description			Temperature, °C		Max ΔT, °C hr^−1^	
Area	2009	No. CTDs	Depths, m	Start, hr	End, hr	Duration, hr	Min/Max	Range	Flood	Ebb
A	Aug 7	12	54–57	−0.43	−0.50	12.22	6.1/11.2	5.1	+1.14	−2.48
B	Aug 8	9	47–50	−0.50	−2.00	10.77	12.3/14.4	2.1	+0.78	−1.62
C	Aug 11	8	44–46	−0.63	−3.23	9.72	9.5/14.3	4.8	+1.65	−1.38
D	Aug 12	8	42–43	−0.35	−3.92	8.77	8.7/14.8	6.1	+0.81	−2.29
E	Aug 13	8	35–37	−2.73	6.10	8.83	14.4/15.6	1.2	+0.73	−0.32

Data at sites were collected during tidal cycles, including at both high and low tide, over periods of 8 to 12 hr. Time-series start and end times are hours before (−) or after (+) high tide. Minimum temperature occurred at low tide (on-bank frontal movement) and maximum temperature occurred at high tide (off-bank frontal movement). Maximum rates (°C hr^−1^) of temperature change (ΔT) were recorded between sequential CTD observations during flood and ebb tidal phases within each time series. See Figures 2,3,4,5,6 for locations of time-series sites (TS) and Figure 8 for temperature cross sections at sites. See Table 4 for temperature data and timing (relative to tidal movement) of the 12 stations at the time-series site in study area A.

Temperature values at the seabed were closely correlated with tidal movement. At all 5 sites, minimum and maximum temperatures occurred at low and high tide, respectively, coincident with on-bank flow of cold gulf water during ebb tide and the off-bank flow of warm bank water during flood tide. Temperature change at the sites during the observation period ranged from 1.2 to 6.1°C and was determined by the site’s location in the frontal zone (Figs. 2,3,4,5,6). Time-series sites in study areas A, C, and D were most affected and recorded temperature changes of 5.1, 4.8, and 6.1°C, respectively; sites in study areas B and E were least affected and recorded temperature changes of 2.1 and 1.2°C, respectively (Table 3).

As expected, seabed temperature did not change uniformly with time (°C hr^−1^) as the tidal front moved across a site. In general, the most rapid rates of temperature change occurred in the middle part of ebb and flood flow when tidal current speeds were predicted to be highest [43]; and the slowest rates of temperature change occurred at high and low tides (slack water) when frontal movement was minimal. At the time-series site in study area A, seabed temperature rate of change (a decrease) was greatest (−2.48°C hr^−1^) approaching mid-ebb flow and then decreased to almost nil (−0.03°C hr^−1^) at low tide (Fig. 8, Table 4). During the subsequent flood tide, the maximum rate of temperature change (an increase) approaching mid flood was much lower (1.14°C hr^−1^). At this site, within a period of approximately 3 hours that included low tide, seabed temperature varied only 0.8°C (6.1 to 6.9°C). Inspection of the time-series temperature-contour plots (Fig. 8) indicates that seabed temperature on the bank changed <1°C in areas A-C during the 3-hour period that bracketed low tide when cold off-bank water was present.

**Table 4:** Hourly rates of seabed temperature change at the time-series site in study area A.

	Timing of observations, hr					Temperature, ^o^C		
CTD sta	Start	Time elapsed	Before Hi	After Hi	Tide stage	T	ΔT	^o^C hr^−1^
052	0.00	–	−0.43		F	9.7	–	–
057	1.32	1.32		0.88	E	11.2	+1.4	+1.09
058	2.25	0.93		1.82	E	11.1	−0.1	−0.10
059	3.38	1.13		2.95	mid ebb	8.3	−2.8	−**2.48**
060	4.37	0.98		3.93	E	6.5	−1.8	−1.83
061	5.33	0.97		4.90	E	6.1	−0.4	−0.36
066	6.55	1.22		6.12	low tide	6.1	0.0	−0.03
069	8.13	1.58	−4.58		F	6.9	+0.8	+0.53
070	9.2	1.07	−3.52		F	8.1	+1.2	**+1.14**
071	10.13	0.93	−2.58		F	8.8	+0.7	+0.71
072	11.07	0.93	−1.65		F	9.1	+0.3	+0.36
073	12.22	1.15	−0.50		F	9.1	0.0	−0.01

Observations were made at intervals ranging from 0.93 to 1.32 hr over a 12.22-hr period. They extended from 0.43 hr before (−) high tide (Hi) to 0.50 hr before (−) high tide and encompassed both flood (F) and ebb (E) stages. Rates of temperature change (^o^C hr^−1^) were calculated using differences in seabed temperature (ΔT) and the time elapsed from the previous observation. Rates were highest at or just after mid-ebb tide (CTD sta 059, −2.48^o^C hr^−1^; CTD sta 060, −1.83^o^C hr^−1^) and just before mid-flood tide (CTD sta 070, 1.14^o^C hr^−1^). Maximum flood and ebb rates of temperature change used for Area A in Table 3 are shown in bold type here. No data were collected at the exact times of high or mid-flood tides, although both were bracketed. Low rates of temperature change bracketed low tide (slack water; CTD sta 061, 066, 069). Temperature decreased during ebb tide (CTD sta 057-061) and increased during flood tide (CTD sta 069-073). See Figure 8 for temperature cross-section at the site.

### 3. Short-term High-frequency Temperature Fluctuations

The CTD data collected between the starts and ends of Seaboss video drifts were not used in mapping the broad-scale effects of frontal movement on seabed temperature in the study region, our interest here. However, these data include phenomena that cannot be observed in CTD profiles of the water column, and examples of them are shown here to illustrate some of the fine-scale hydrographic complexity of the frontal structure. The video drift data need to be viewed with some caution as they represent records collected from a moving vehicle (25–100 cm s^−1^).

In warm well-mixed water in on-bank subareas (Fig. 9A), temperature patterns showed <0.1°C overall temperature change, and included high frequency fluctuations of <0.01°C with periods of <2 s. Within an hour of low tide (slack water), in both off-bank and on-bank subareas (Fig. 9B), small temperature changes were observed that ranged up to 0.02°C with periods up to 10 s. Within the 2- to 5-hour period bracketing high tide, large variations in temperature were recorded in off-bank subareas (Fig. 9C). Temperature fluctuations had amplitudes of up to 0.1°C and periods of up to 10 s, and overall temperature changes ranged up to 0.5°C, with rates of up to 2°C hr^−1^. In off-bank subareas, several patterns of rapid temperature fluctuations occurred, including single rapid changes, up to 0.6°C in 0.2 min (Fig. 10A); and multiple saw tooth fluctuations of temperature in rapid succession, some up to 0.3°C in 4 to 5 min (Fig. 10B), and some up to 2–3°C in 6 min (Fig. 10C).

### 4. Areal and Temporal Distribution of Seabed Temperatures Below the Tidal Front

Variation in seabed temperatures in study areas was chiefly in on- and off-bank directions (parallel to tidal flow and frontal movement). Temperature data collected at sites along transects, at time-series sites, and from the starts and ends of Seaboss video drift deployments were not evenly distributed within the study areas (Fig. 1). Nevertheless, plotting of temperature data collected during flood and ebb tides at sites within each study area revealed the presence of geographic subareas (6 to 8 in each study area; Figs. 2,3,4,5,6) with distinctive temperature-change characteristics (Fig. S1A-E, Tables 5, S3). Data from study area E are not discussed in detail here because its off-bank part extended only to 60 m water depth and was sparsely sampled. Its on-bank part (subareas 4–6; Fig. S1E) showed seabed temperature characteristics similar to on-bank subareas of study areas A-D described below.

**Table 5:** Summary of seabed water depths and the frontal effect on temperature change in subareas of study areas A–E.

			Depth, m		Temperature, °C	
Area, subarea	Width, km	CTD sta	Min/Max	Range	Min/Max	Range
A,1	5.5	74	52/87	35	5.7/12.2	6.5
A,2	2.7	28	49/56	7	6.7/13.6	6.9
A,3	3.0	33	46/58	12	7.9/13.9	6.0
A,4	2.7	11	48/56	8	9.8/13.8	4.0
A,5	2.7	6	53/56	3	12.4/13.8	1.4
A,6	7.3	6	52/56	4	13.4/14.2	0.8
A,7	5.2	6	51/59	8	14.0/14.2	0.2
B,1	3.1	5	62/82	20	6.1/10.0	3.9
B,2	3.0	8	50/54	4	6.9/13.2	6.3
B,3	2.6	9	51/52	1	6.7/13.6	6.9
B,4	3.0	20	46/55	9	8.7/14.6	5.9
B,5	4.1	37	46/55	9	12.3/14.7	2.4
B,6	6.2	20	48/58	10	13.5/14.3	0.8
B,7	6.4	8	47/51	4	14.1/14.3	0.2
C,1	3.1	4	64/81	17	5.5/11.2	5.7
C,2	2.7	6	48/51	3	7.4/14.2	6.8
C,3	2.8	21	43/47	4	9.1/14.3	5.2
C,4	2.9	11	43/46	3	10.4/14.4	4.0
C,5	2.6	6	46/50	4	12.8/14.4	1.6
C,6	2.8	4	50/53	3	13.1/14.7	1.6
C,7	6.3	11	46/67	11	13.8/15.0	1.2
C,8	9.8	7	54/59	5	14.8/15.1	0.3
D,1	2.2	4	86/94	8	5.1/7.9	2.8
D,2	2.4	8	60/76	16	5.3/13.2	7.9
D,3	3.0	10	42/59	17	5.8/12.5	6.7
D,4	3.0	14	40/47	7	7.1/15.1	8.0
D,5	2.8	4	37/42	5	11.3/15.7	4.4
D,6	2.7	4	37/39	2	13.8/15.7	1.9
D,7	2.6	6	38/39	1	14.3/16.0	1.7
D,8	7.3	6	37/45	8	15.1/15.7	0.6
E,1	0.5	5	59/60	1	8.4/12.1	3.7
E,2	3.3	4	40/47	7	11.1/12.9	1.8
E,3	2.9	6	37/41	4	12.1/15.3	3.2
E,4	4.4	34	27/38	11	13.4/15.7	2.3
E,5	2.6	14	30/35	5	14.8/16.3	1.5
E,6	4.2	4	32/34	2	15.5/16.4	0.9

**Figure 11:**
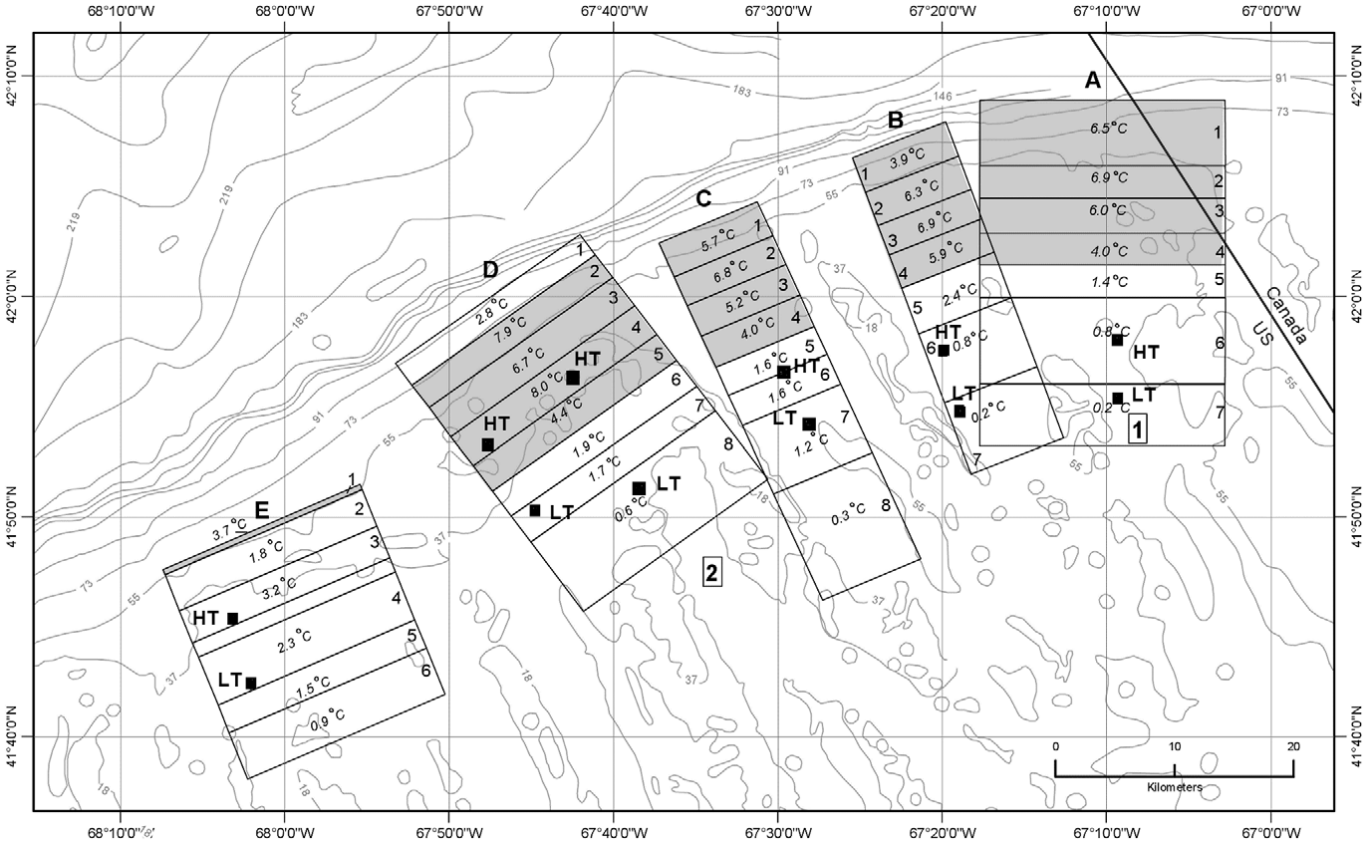
Map of study areas A-E and numbered subareas with tidal temperature changes. Values in each subarea are changes in seabed temperature (°C) during tidal front movement off bank (flood tide) and on bank (ebb tide) during the August 4–13, 2009 time period. Shaded subareas represent regions of greatest effect on seabed temperature (40 to 80 m depth interval). Unshaded subareas represent regions of lesser tidal effect on temperatures (see Tables 5, S3 for details). The frontal zone is the area where seabed temperature is changed >1°C by tidal movement of the front. Square black markers indicate the approximate positions of the frontal boundary (i.e. the transition from a stratified to a mixed water column) along each of the linear transects during high tide (HT) and low tide (LT). The area between HT and LT is the frontal boundary zone. Numbers in rectangles and bathymetry are as in Fig. 1.

Temperature data was collected at CTD stations located along transects, at time-series stations, and at the starts and ends of video-drift stations. Subareas in each study area are numbered in an off-bank to on-bank direction (Figs. 2,3,4,5,6). Subarea widths are measured normal to the tidal front. The range of seabed temperatures experienced by each subarea is shown and indicates which subareas are affected by frontal movement. The largest temperature changes between high and low tide (ranging from 3.9 to 8.0^o^C) occurred in subareas that lie in a 11–14 km wide band that extends ∼100 km along the bank margin above 80 m water depth (Fig. 11). Table S3 expands this data by showing the frontal effect on temperature change in the subareas during flood and ebb tides.

Seabed temperatures in off-bank subareas followed the tidal pattern and tended, with few exceptions (Fig. S1A, subarea 2), to reach their maxima and minima within an hour of high and low tide, respectively. In those on-bank subareas where the water column was mixed, temperatures were nearly isothermal (Fig. S1 C, subarea 8). In off-bank subareas, the most northerly (subareas 1 in study areas A-D) displayed low minimum temperatures due to the strong influence of cold off-bank water that moved onto the bank during ebb tide, and relatively low maximum temperatures due to the weak influence of warm on-bank water that moved off the bank during flood tide (Fig. S1A-D). In other subareas (2 and higher) of the study areas, both temperature minima and maxima increased in the on-bank direction in response to the increasing influence of warm on-bank water during flood tide and the decreasing influence of cold off-bank water during ebb tide.

The geographic movement and associated temperature effect of the tidal front were approximated by determining the magnitude of temperature change occurring in a subarea, based on all temperature observations from that subarea. In many of the more northerly subareas, seabed temperatures changed more than 3°C as a result of tidal movement (Table 5). In study area A (subareas 1–4), temperature change ranged from 4.0 to 6.9°C; in study area B (subareas 1–4), temperature change was 3.9 to 6.9°C; in C (subareas 1–4), 4.0 to 6.8°C; and in D (subareas 2–5), 4.4 to 8.0°C (Fig. S1A-D). In some subareas, these data were collected over a 12-hour period during both flood and ebb tides, while in others data were collected only during flood or ebb tides. The overall pattern of temperature variation indicates that frontal movement caused significant temperature changes in small geographic areas during a 6-hour flood or ebb tide. By contrast, in the more southerly on-bank subareas, where warm mixed on-bank water dominated, minima and maxima showed little difference and the temperature change was <1°C (Fig. S1A-D).

Subareas experiencing maximum temperature change occurred in the frontal zone but not in the frontal boundary zone (Fig. 11). Rather they were located several km seaward (off-bank) of the frontal boundary zone in areas A, B, and C; and partially overlapped the frontal boundary zone in area D.

Temperature dynamics along the bank margin were very similar in study areas A-D. The subareas most affected by temperature change (3.9 to 8.0°C) during frontal movement extended approximately from 40 m on the bank to 80 m on the bank edge (Fig. 11, Table 5). With increasing distance off the bank and increasing water depth in area D, the front had reduced effect on seabed temperatures. The deepest part of area D (subarea 1, 86–94 m) exhibited a much-reduced temperature change of 2.8°C compared with the next shallower interval (subarea 2, 60–76 m) where the temperature change was 7.9°C. We assume if study areas A-C were extended northward off the bank, they also would show this trend of declining temperature change with increasing water depth. Individual subareas showing the greatest temperature change at the seabed were located at depths of 40 to 56 m and included: study area A (subarea 2), 6.9°C, depth 49–56 m; area B (subarea 3), 6.9°C, 51–52 m; area C (subarea 2), 6.8°C, 48–51 m; and area D (subarea 4), 8.0°C, 40–47 m (Table 5). In area E, sampling along transect T24 (Fig. 6) did not extend below 60 m and temperature change in its most affected subarea was 3.7°C.

## Summary and Discussion

### 1 Tidal Front Movement and Seabed Temperature

In summer on the northern margin of Georges Bank, a tidal-mixing front forms between the thermally stratified off-bank water of the Gulf of Maine to the north and mixed on-bank water to the south. At the seabed, cool water from the gulf and warm water from the bank move back and forth in the frontal zone in response to movements of the semidiurnal tide. During our study in August 2009, gulf water was warm (∼18°C) at the surface and cold (∼6°C) at the seabed at water depths of 80 to 90 m. In contrast, bank water displayed a relatively uniform temperature that varied <1°C in the range of 14 to 16°C from the surface to the seabed at depths of 40 to 60 m. The semidiurnal movement of the tidal-mixing front that formed where these very different water masses interacted affected the entire length (∼100 km) of our study region. Our analyses, and those of previous studies ([18]: Fig. 7A-F), show that the movement of the tidal-mixing front in summer subjects seabed habitats beneath it to changes in temperature four times a day.

Water column temperature data collected along 6 transects across the bank margin in 5 study areas showed that the frontal boundary moved 6 to 10 km between high and low tides in August 2009 (Fig. 7A-F), in line with observations in July 1988 by Loder et al. ([18]: Fig. 7A-F). Observations of seabed temperature along our transects showed that temperature change at sites with paired stations (high and low tide) ranged from 0.0 to 7.0°C. Along the parts of transects affected by the front, temperatures changed with distance on average 0.21 to 0.55°C km^−1^ in the on- and off-bank directions (Table 2,S2). The spacing of isotherms at the seabed indicated that variations in temperature were not uniform across the frontal zone (Fig. 7A-F). Most cross sections of the frontal structure displayed localized depressions in isotherms of 10 to 40 m in depth (Fig. 7A-E), suggesting the presence of internal wave-like phenomena in the water column as described by Loder, et al. ([18]: Figs. 9, 10). These appeared at both high and low tide, presumably reflecting their development during both flood and ebb tide intervals, as reported by these authors.

Water column time-series temperature data collected at individual sites showed that the magnitude of seabed temperature change caused by tidal movement was variable in the frontal zone. Maximum temperature change at the seabed ranged from 1.2 to 6.1°C at the 5 sites and was influenced by geographic location. The magnitude of change was greatest in the 40 to 80 m depth interval, and seabed temperatures changed with time as much as 2.48°C hr^−1^ (Table 3). The rate of change was greatest at mid-ebb and mid-flood tide, when tidal currents were predicted to be strongest, with little change in temperature in the 3 hours bracketing slack water (Fig. 8).

CTD data collected along Seaboss video drift tracks in the frontal zone provided evidence for short-term temperature instability at the seabed. High frequency fluctuations (period ≤20 seconds) occurred to a varying degree in subareas affected by frontal movement (Fig. 9C) but not in subareas with a mixed water column (Fig. 9A). The video drift data also exhibited sharp temperature fluctuations and cyclic variations (2 to 3°C) that strongly suggest the passage of internal waves (Fig. 10A-C).

Analyses of seabed CTD temperature data from transect and time-series stations, and the starts and ends of video drift stations, revealed that the geographic extent of the frontal effect on the seabed in 5 study areas can be mapped as subareas with distinctive temperature-change characteristics (Fig. 11). Within each subarea, the tidally-induced temperature change at the seabed was approximated by basing it on the aggregation of all temperature observations from the subarea. This approach revealed geographic areas of the bank margin that were characterized by temperature stability or instability depending on location and tidal dynamics. Subareas within each of the 5 study areas experienced temperature changes in the range of 0.2 to 8.0°C between high and low tides (Table 5, S3). Temperature changes caused by frontal movement decreased with both increasing water depth in the off-bank direction and decreasing depth in the on-bank direction. In the deepest subarea (area D, subarea 1; 86–94 m), temperatures at the seabed remained relatively cold (5.1–7.9°C) from high to low tide. Similarly, at relatively shallow depths on the bank (area D, subarea 8; 37–45 m) temperatures remained relatively warm (15.1–15.7°C) during a like period (Table 5, S3). The largest temperature changes attributed to frontal movement ranged from 3.9 to 8.0°C in subareas of study areas A-D and occurred along the bank margin in a 15- to 18 km-wide band in the 40 to 80 m depth interval (Fig. 11). In area E, the effect of frontal movement on seabed temperature is not well documented, but existing data suggest temperature change was largest in water depths of 60 m and deeper.

### 2 Ecological Implications for the Fishery

The large tidally-driven temperature changes within the frontal zone on the northern margin of Georges Bank in August likely have ecological implications for fish and invertebrate species that inhabit the region. The northern margin exhibits the highest benthic production on an otherwise highly productive bank [5], much of it attributable to sea scallops (*Placopecten magellanicus*) [4]. Yet this productivity may come with a high physiological and/or ecological price. Benthic invertebrates must endure rapid and substantial temperature changes during each flood and ebb tide. In some locations, temperatures vary as much as 7.0°C (Table 1) over a 6-hour period at short-term rates of up to 2.5°C hr^−1^. They may also experience very short-term temperature instabilities associated with turbulent mixing. Fish conceivably can respond directly to temperature changes by moving horizontally and/or vertically to remain within their zone of acclimation. There is evidence from the scientific literature (see below) that cyclic temperature changes have an effect on the behavior and distribution of invertebrate and fish species, although no such studies have been conducted in a region of the continental shelf where temperatures are driven by movements of a tidal-mixing front.

Large, rapid, cyclical swings in temperature are uncommon in most shelf environments, but they are typical of intertidal and shallow subtidal settings where it is well documented that tidally-driven temperature changes result in zonation of resident species [46]–[48]. On the northern margin of Georges Bank one might expect to find that cyclical temperature changes similarly affect the distribution of resident species and result in a zonation of benthic communities. Such zonation patterns have not been reported from the frontal zone, possibly because their detection has been confounded by the heterogeneity of surficial sediments there [49], by the effects of disturbance by bottom fishing [50], and by the lack of directed study.

Cyclic temperatures have been shown in laboratory experiments to affect the growth, and development of larval and adult crustaceans from freshwater and coastal environments including mud crab larvae [51], shrimp [52] and juvenile crayfish [53]. These results are suggestive of the effects that may be imposed on early life history stages of seabed animals by cyclic temperature regimes, but are not directly applicable to our study area because they were not conducted on species or in temperature and salinity conditions typical of the Georges Bank frontal zone. In a study of summer flounder larvae (*Paralichthys dentatus*), a species that occurs on Georges Bank, Johns et al. [54] measured their development rates in a variety of constant and cyclic temperature regimes and found that larvae raised in cyclic temperatures developed faster than those raised at a constant temperature of 5°C, but slower than those raised at 21°C. Larvae reared at the coldest constant temperature (5°C) and those reared in the coldest cyclic temperature regime (5–11°C) did not survive. Summer flounder eggs, larvae, and adults are present on Georges Bank but are sparse in the frontal zone [55].

A study of the American lobster (*Homarus americanus*), a resident of Georges Bank and the frontal zone, showed that for individuals acclimated to temperatures of 2, 5, 10, 15, 20, or 25°C, walking activity was lowest at 2 and 5°C and generally increased as acclimation temperatures increased [56]. When lobsters from these acclimation groups were tested after being subjected to a rapid change in temperature for at least 1 hour, those acclimated to 2 and 5°C were most active at 5–10°C, those acclimated to 10°C were most active at 15°C, and those acclimated to 15, 20, or 25°C were most active at the acclimation temperature. In addition, lobsters acclimated to 15°C were inactive at 2°C, those acclimated to 20°C were inactive at 5°C, and those acclimated to 25°C were inactive at 10°C. These results suggest that tidally-driven, cyclic temperature changes possibly regulate lobster activity by slowing it during ebb tide when cool water moves southward onto the bank and increasing it during flood tide when flow is reversed and warm bank water moves northward across the bank margin.

Claireaux et al. [57] conducted a study of the behavioral response of Atlantic cod (*Gadus morhua*) acclimated at 5°C to temperatures in the range of 4 to 11°C. They reported that in a mixed water column in a test tank, voluntary swimming activity was doubled by a rise in temperature of 2°C (to 7°C). In a stratified water column, cod avoided cold (4°C) water introduced at the base of the tank; and the fish preferred deeper, colder water during the day and shallow, warmer water at night. These observations suggest that Atlantic cod, a major fishery species of the bank, possibly exhibit a behavioral response to the temperature changes caused by movement of the tidal-mixing front.

Lough [58] has shown that survival of recently-settled (July) juvenile Atlantic cod is enhanced in the gravel habitat of northern Georges Bank, part of which lies in our study region. He suggested that complex gravel habitat serves the cod both as a source of prey and as a refuge from predators. The distribution of juvenile cod can now also be examined to determine if the tidally-induced seabed temperature changes described herein affect their behavior and habitat preferences.

We speculate that the response of fish and invertebrates to tidally-induced, cyclic temperature changes in the frontal zone depends on how species are affected by the temperature change between high and low tide, by the rate of temperature change with time, and by the duration of temperature stability at high and low tide. A number of questions are raised by our analysis of temperature cycles in the frontal zone. 1. Do demersal fish species follow the front during flood and ebb tides to maintain their acclimated temperature and/or to gain access to feeding grounds at temperatures favorable to them; or do they remain within an area and alter their physiology and behavior in response to *in situ* temperature changes? 2. How is the activity level of mobile invertebrates affected by cyclic temperature changes at the seabed? 3. Do temperature fluctuations in the frontal zone affect the survival and/or reproduction of benthic invertebrate species and prevent some species from living there; thus influencing community composition in parts of a large region of gravel substrate? 4. Does alternating occupation of the frontal zone by water masses from Georges Bank and the Gulf of Maine provide, over periods of hours, different kinds and quantities of food for filter-feeding invertebrate species, thus affecting local benthic productivity?

We anticipate that the observations reported here will provide a framework for ecological studies to determine the effects of short-term temperature variability on the distribution, behavior, and food and shelter resources of both invertebrate and fish species that inhabit the northern margin of Georges Bank in summer when the front is well developed. Additionally, these results should be helpful in calibrating hydrographic models for predicting seabed temperature patterns in areas of the bank affected by the tidal-mixing front.

## Supporting Information

Figure S1 Seabed temperature versus tidal phase. Seabed temperature is plotted against time before (−) and after (+) high tide in the subareas of study areas A-E on the northern margin of Georges Bank in August 2009. Subareas are numbered as in Figs. 2–6. A pattern of larger changes in the deeper subareas within each area is evident, as summarized in Fig. 11.
(PDF)

Table S1 Water depth, seabed temperature, and temperature change along transects across the tidal front. Temperature change from high to low tide is shown for paired CTD stations at observation sites along transects in study areas A-E. Station pairs are grouped in Table 1. In each study area, station pairs (column 1) are numbered from north to south (i.e. off bank to on bank). Individual stations (columns 2–3) are numbered 001-214. The change in temperature between high and low tides at the paired stations is Hi-Lo ΔT. By our definition, a site is affected (Y) by frontal movement if Hi-Lo ΔT is >1.0°C. See Figure 1 for locations of study areas; Figures 2,3,4,5,6 for locations of CTD transects; and Figure 7 for locations of individual CTD stations.
(DOC)

Table S2 Comparison of parts of seabed transects along which temperature is affected or not affected by the tidal front at high and low tide. Stations along each transect are arranged across the tidal front from deep to shallow (off bank to on bank). Seabed temperature change per kilometer is given for parts of transects where individual sites are affected or not affected by the tidal front. By our definition, a site is affected by frontal movement if the temperature change between high and low tides is >1°C (Table S1, Hi-Lo ΔT). For example, in study area A at high tide, the part of transect T16 affected by the front extended from station 026 to station 031 over a distance of 17.1 km. Water depths at 026 and 031 were 79 and 54 m, respectively. Temperature changed from a minimum of 9.8°C at station 026 (in the deep part) to a maximum of 13.6°C at station 031 (in the shallow part). Thus at high tide, temperature changed 3.8°C over 17.1 km and the rate of change was 0.22°C km^−1^. In area D, all stations along transect 23 were affected by frontal movement. See Table S1 for depth and temperature data for all stations. See Figure 7 for locations of stations along CTD transects.
(DOC)

Table S3 Summary of seabed water depths and the frontal effect on temperature change in subareas of study areas A-E. Temperature data was collected at numerous CTD stations located along transects, at time-series stations, and at the starts and ends of video-drift stations. Subareas in each study area are numbered in an off-bank to on-bank direction (Figs. 2,3,4,5,6). Subarea widths are measured normal to the tidal front. Temperature data collected during flood or ebb tides are plotted in Figure S1. The largest temperature changes between high and low tide (ranging from 3.9 to 8.0°C) occurred in subareas that lie in a 11–14 km wide band that extends ∼100 km along the bank margin above 80 m water depth (Fig. 11). Abbreviations: nd – no data; ndef - data not definitive to determine range.
(DOC)
