# Supplementary figures and images for: Semidiurnal Temperature Changes Caused by Tidal Front Movements in the Warm Season in Seabed Habitats on the Georges Bank Northern Margin and Their Ecological Implications
Source: PLoS One. 2013 Feb 6;8(2):e55273. doi: 10.1371/journal.pone.0055273 (PMC3566201; doi:10.1371/journal.pone.0055273)

A

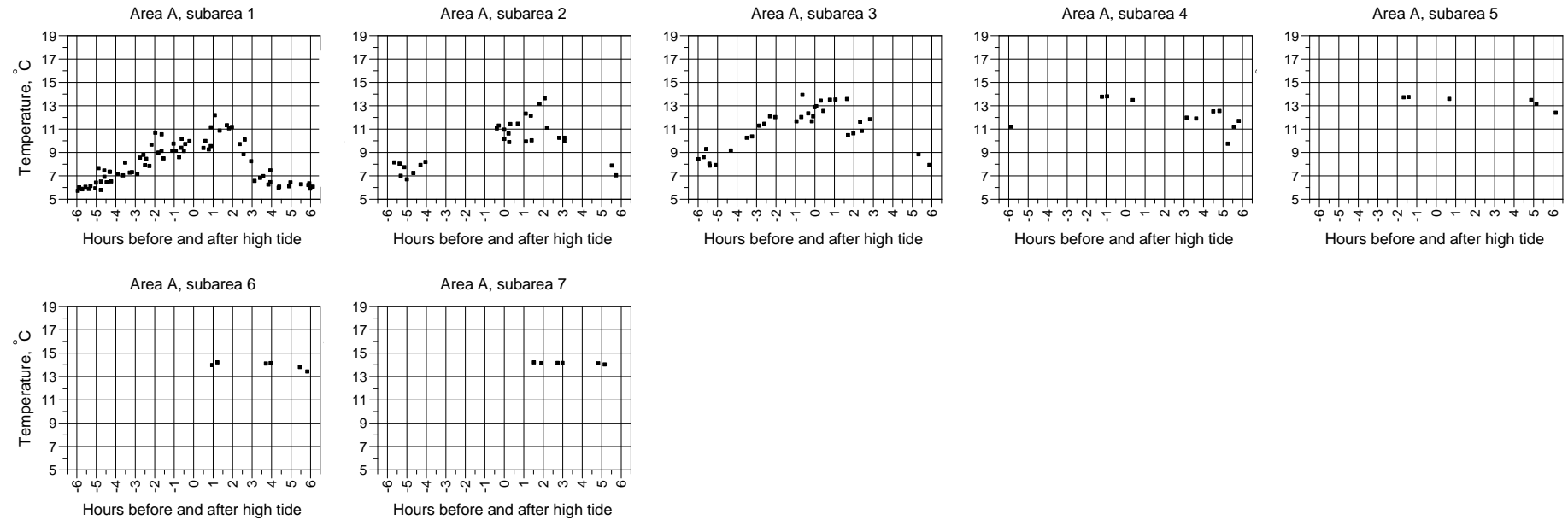

B

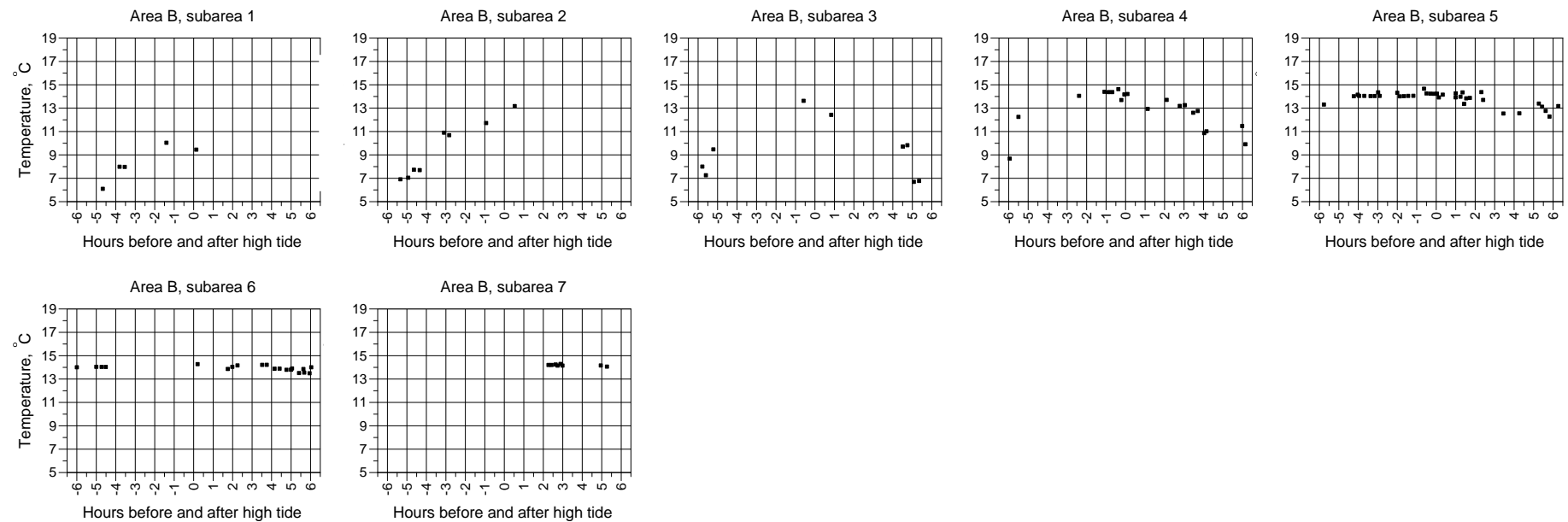

C

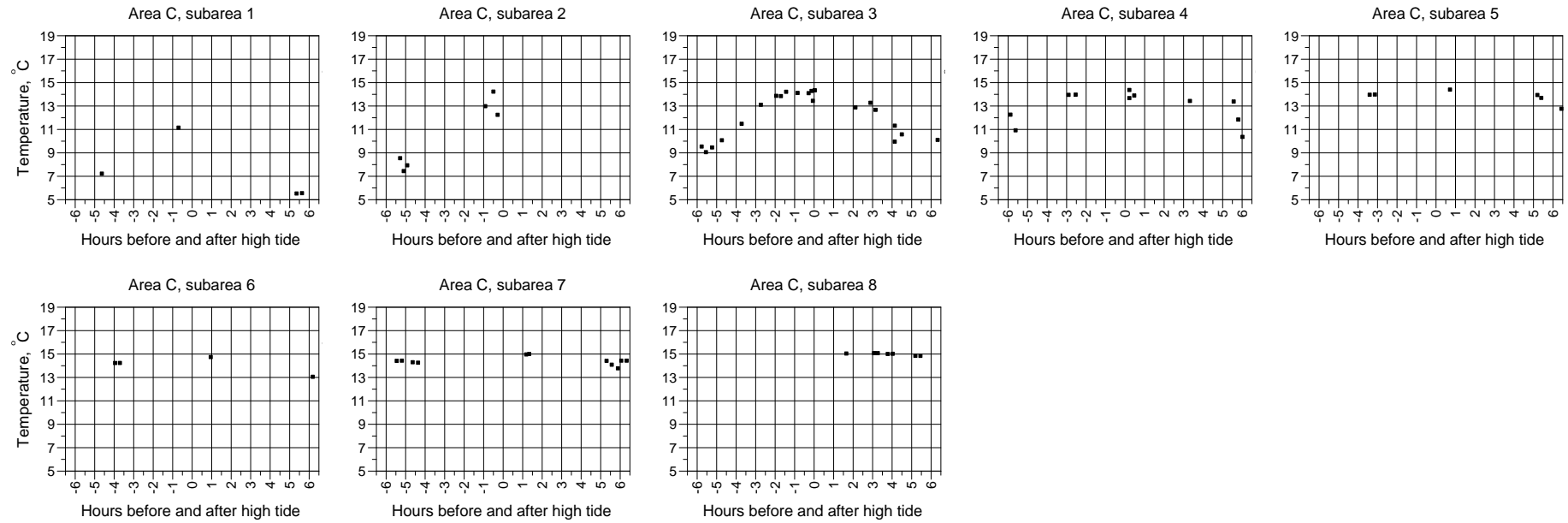

D

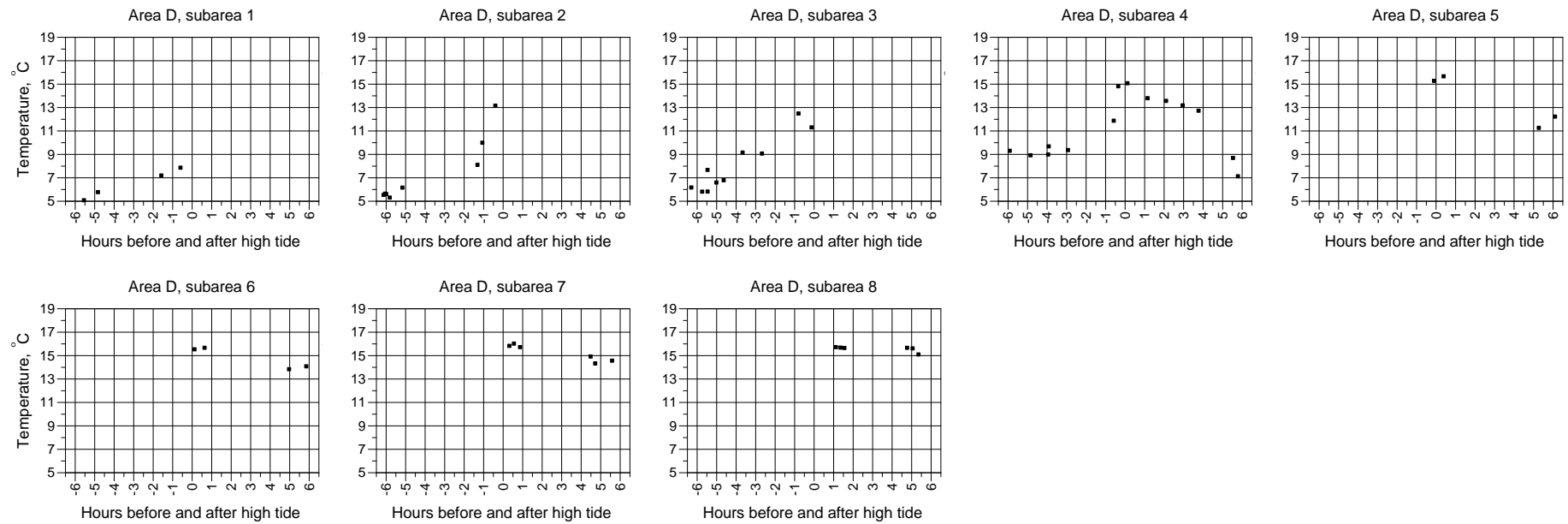

E

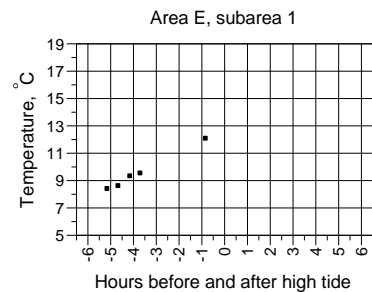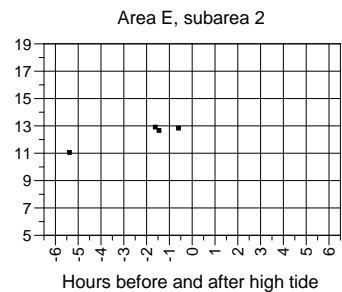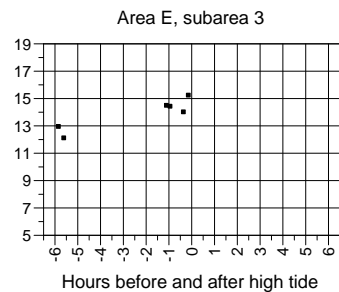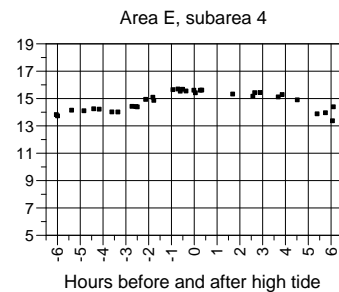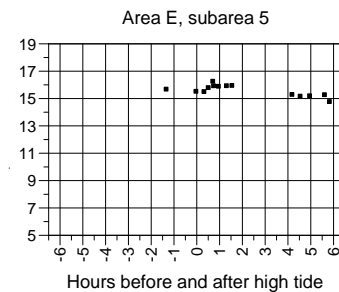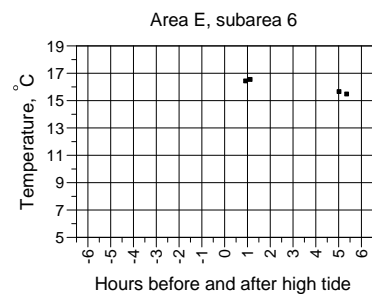

Supplement: Figure S1 — Seabed temperature versus tidal phase. Seabed temperature is plotted against time before (−) and after (+) high tide in the subareas of study areas A-E on the northern margin of Georges Bank in August 2009. Subareas are numbered as in Figs. 2–6. A pattern of larger changes in the deeper subareas within each area is evident, as summarized in Fig. 11. (PDF) [file pone.0055273.s004.doc]
