# Supplementary material for: Semidiurnal Temperature Changes Caused by Tidal Front Movements in the Warm Season in Seabed Habitats on the Georges Bank Northern Margin and Their Ecological Implications
Source: PLoS One. 2013 Feb 6;8(2):e55273. doi: 10.1371/journal.pone.0055273 (PMC3566201; doi:10.1371/journal.pone.0055273)
Supplement: Table S1 — Water depth, seabed temperature, and temperature change along transects across the tidal front. Temperature change from high to low tide is shown for paired CTD stations at observation sites along transects in study areas A-E. Station pairs are grouped in Table 1. In each study area, station pairs (column 1) are numbered from north to south (i.e. off bank to on bank). Individual stations (columns 2–3) are numbered 001-214. The change in temperature between high and low tides at the paired stations is Hi-Lo ΔT. By our definition, a site is affected (Y) by frontal movement if Hi-Lo ΔT is >1.0°C. See Figure 1 for locations of study areas; Figures 2,3,4,5,6 for locations of CTD transects; and Figure 7 for locations of individual CTD stations. (DOC) [file pone.0055273.s001.doc]

| Location | Station | Stations | | Depth, m | | Temperature, ^o^C | | | Frontal |
| --- | --- | --- | --- | --- | --- | --- | --- | --- | --- |
| Area, transect | pair | Hi tide | Lo tide | Hi tide | Lo tide | Hi tide | Lo tide | Hi-Lo ΔT | effect |
| A, T16 | 1 | 026 | 045 | 79 | 81 | 9.8 | 5.8 | 4.0 | Y |
|  | 2 | 027 | 044 | 57 | 56 | 10.2 | 5.9 | 4.3 | Y |
|  | 3 | 028 | 043 | 52 | 51 | 11.3 | 7.0 | 4.3 | Y |
|  | 4 | 029 | 042 | 53 | 52 | 13.0 | 9.3 | 3.7 | Y |
|  | 5 | 030 | 041 | 55 | 54 | 13.5 | 11.2 | 2.3 | Y |
|  | 6 | 031 | 040 | 54 | 53 | 13.6 | 12.4 | 1.2 | Y |
|  | 7 | 032 | 039 | 53 | 54 | 14.0 | 13.4 | 0.6 | N |
|  | 8 | 033 | 038 | 56 | 55 | 14.2 | 13.8 | 0.4 | N |
|  | 9 | 034 | 037 | 52 | 53 | 14.2 | 14.0 | 0.2 | N |
|  | 10 | 035 | 036 | 59 | 57 | 14.1 | 14.1 | 0.0 | N |
| B, T19 | 1 | 001 | 020 | 82 | 82 | 9.5 | 6.1 | 3.4 | Y |
|  | 2 | 002 | 019 | 53 | 52 | 13.2 | 7.1 | 6.1 | Y |
|  | 3 | 003 | 018 | 51 | 51 | 12.4 | 9.5 | 2.9 | Y |
|  | 4 | 004 | 017 | 50 | 49 | 12.9 | 12.3 | 0.6 | N |
|  | 5 | 005 | 016 | 53 | 52 | 13.4 | 13.3 | 0.1 | N |
|  | 6 | 006 | 015 | 57 | 55 | 13.9 | 13.2 | 0.7 | N |
|  | 7 | 007 | 014 | 53 | 54 | 14.0 | 13.5 | 0.5 | N |
|  | 8 | 008 | 013 | 50 | 48 | 14.2 | 13.9 | 0.3 | N |
|  | 9 | 009 | 012 | 50 | 50 | 14.2 | 14.1 | 0.1 | N |
|  | 10 | 010 | 011 | 48 | 47 | 14.3 | 14.2 | 0.1 | N |
| C, T18 | 1 | 100 | 119 | 68 | 64 | 11.2 | 7.2 | 4.0 | Y |
|  | 2 | 101 | 118 | 49 | 51 | 12.3 | 7.9 | 4.4 | Y |
|  | 3 | 102 | 117 | 46 | 45 | 13.4 | 9.5 | 3.9 | Y |
|  | 4 | 103 | 116 | 44 | 43 | 13.7 | 10.9 | 2.8 | Y |
|  | 5 | 104 | 115 | 46 | 45 | 13.9 | 12.3 | 1.6 | Y |
|  | 6 | 105 | 114 | 47 | 46 | 14.4 | 12.8 | 1.6 | Y |
|  | 7 | 106 | 113 | 52 | 50 | 14.7 | 13.1 | 1.6 | Y |
|  | 8 | 107 | 112 | 64 | 63 | 15.0 | 13.8 | 1.2 | Y |
|  | 9 | 108 | 111 | 60 | 59 | 15.0 | 14.1 | 0.9 | N |
|  | 10 | 109 | 110 | 54 | 52 | 15.0 | 14.4 | 0.6 | N |
| D, T22 | 1 | 120 | 139 | 88 | 86 | 7.9 | 5.8 | 2.1 | Y |
|  | 2 | 121 | 138 | 60 | 60 | 13.2 | 6.2 | 7.0 | Y |
|  | 3 | 122 | 137 | 50 | 42 | 11.3 | 7.7 | 3.6 | Y |
|  | 4 | 123 | 136 | 43 | 42 | 15.1 | 9.3 | 5.8 | Y |
|  | 5 | 124 | 135 | 39 | 37 | 15.7 | 12.2 | 3.5 | Y |
|  | 6 | 125 | 134 | 39 | 37 | 15.7 | 14.1 | 1.6 | Y |
|  | 7 | 126 | 133 | 39 | 38 | 15.7 | 14.6 | 1.1 | Y |
|  | 8 | 127 | 132 | 39 | 38 | 15.7 | 15.1 | 0.6 | N |
|  | 9 | 128 | 131 | 43 | 37 | 15.7 | 15.6 | 0.1 | N |
|  | 10 | 129 | 130 | 45 | 45 | 15.7 | 15.7 | 0.0 | N |
| D, T23 | 1 | 163 | 186 | 92 | 94 | 7.2 | 5.1 | 2.1 | Y |
|  | 2 | 164 | 185 | 76 | 75 | 8.1 | 5.3 | 2.8 | Y |
|  | 3 | 165 | 184 | 61 | 61 | 10.0 | 5.7 | 4.3 | Y |
|  | 4 | 166 | 183 | 50 | 52 | 12.5 | 6.2 | 6.3 | Y |
|  | 5 | 167 | 182 | 45 | 40 | 11.9 | 7.1 | 4.8 | Y |
|  | 6 | 168 | 181 | 43 | 42 | 14.8 | 8.7 | 6.1 | Y |
|  | 7 | 169 | 180 | 42 | 41 | 15.3 | 11.3 | 4.0 | Y |
|  | 8 | 170 | 179 | 38 | 37 | 15.5 | 13.8 | 1.7 | Y |
|  | 9 | 171 | 178 | 39 | 38 | 15.8 | 14.3 | 1.5 | Y |
|  | 10 | 172 | 177 | 39 | 38 | 16.0 | 14.9 | 1.1 | Y |
| E, T24 | 1 | 191 | 214 | 60 | 59 | 12.1 | 8.4 | 3.7 | Y |
|  | 2 | 192 | 213 | 47 | 46 | 12.8 | 11.1 | 1.7 | Y |
|  | 3 | 193 | 212 | 40 | 38 | 14.0 | 12.1 | 1.9 | Y |
|  | 4 | 194 | 211 | 41 | 40 | 15.3 | 13.0 | 2.3 | Y |
|  | 5 | 195 | 210 | 38 | 36 | 15.4 | 13.8 | 1.6 | Y |
|  | 6 | 196 | 209 | 37 | 36 | 15.6 | 14.4 | 1.2 | Y |
|  | 7 | 197 | 208 | 35 | 34 | 15.8 | 14.8 | 1.0 | N |
|  | 8 | 198 | 207 | 35 | 34 | 16.3 | 15.3 | 1.0 | N |
|  | 9 | 199 | 206 | 34 | 32 | 16.4 | 15.5 | 0.9 | N |
|  | 10 | 200 | 205 | 33 | 32 | 16.3 | 15.7 | 0.6 | N |

Table S1. Water depth, seabed temperature, and temperature change along transects across the tidal front.
