# Supplementary material for: Semidiurnal Temperature Changes Caused by Tidal Front Movements in the Warm Season in Seabed Habitats on the Georges Bank Northern Margin and Their Ecological Implications
Source: PLoS One. 2013 Feb 6;8(2):e55273. doi: 10.1371/journal.pone.0055273 (PMC3566201; doi:10.1371/journal.pone.0055273)
Supplement: Table S2 — Comparison of parts of seabed transects along which temperature is affected or not affected by the tidal front at high and low tide. Stations along each transect are arranged across the tidal front from deep to shallow (off bank to on bank). Seabed temperature change per kilometer is given for parts of transects where individual sites are affected or not affected by the tidal front. By our definition, a site is affected by frontal movement if the temperature change between high and low tides is >1°C (Table S1, Hi-Lo ΔT). For example, in study area A at high tide, the part of transect T16 affected by the front extended from station 026 to station 031 over a distance of 17.1 km. Water depths at 026 and 031 were 79 and 54 m, respectively. Temperature changed from a minimum of 9.8°C at station 026 (in the deep part) to a maximum of 13.6°C at station 031 (in the shallow part). Thus at high tide, temperature changed 3.8°C over 17.1 km and the rate of change was 0.22°C km−1. In area D, all stations along transect 23 were affected by frontal movement. See Table S1 for depth and temperature data for all stations. See Figure 7 for locations of stations along CTD transects. (DOC) [file pone.0055273.s002.doc]

|  |  |  | Part of CTD transect AFFECTED by tidal front | | | | | | Part of CTD transect NOT AFFECTED by tidal front | | | | | |
| --- | --- | --- | --- | --- | --- | --- | --- | --- | --- | --- | --- | --- | --- | --- |
| Location |  |  |  |  |  | Temperature, °C | | |  |  |  | Temperature, °C | | |
| Area, transect | Tide | Transect, km | Stations | Dist., km | Depth, m | Min/Max | ΔT | °C km^-1^ | Stations | Dist., km | Depth, m | Min/Max | ΔT | °C km^-1^ |
| A, T16 | Hi | 27.9 | 026-031 | 17.1 | 79-54 | 9.8/13.6 | 3.8 | 0.22 | 032-035 | 10.8 | 53-59 | 14.0/14.1 | 0.1 | 0.01 |
| A, T16 | Lo | 27.8 | 045-040 | 17.1 | 81-53 | 5.8/12.4 | 6.6 | 0.39 | 039-036 | 10.8 | 54-57 | 13.4/14.1 | 0.7 | 0.07 |
| B, T19 | Hi | 27.1 | 001-003 | 7.2 | 82-51 | 9.5/12.4 | 2.9 | 0.40 | 004-010 | 19.9 | 50-48 | 12.9/14.3 | 1.4 | 0.07 |
| B, T19 | Lo | 27.2 | 020-018 | 7.7 | 82-51 | 6.1/9.5 | 3.4 | 0.44 | 017-011 | 19.6 | 49-47 | 12.3/14.2 | 1.9 | 0.10 |
| C, T18 | Hi | 21.1 | 100-107 | 18.5 | 68-64 | 11.2/15.0 | 3.8 | 0.21 | 108-109 | 2.6 | 60-54 | 15.0/15.0 | 0.0 | 0.00 |
| C, T18 | Lo | 21.5 | 119-112 | 18.0 | 64-63 | 7.2/13.8 | 6.6 | 0.37 | 111-110 | 3.5 | 59-52 | 14.1/14.4 | 0.3 | 0.09 |
| D, T22 | Hi | 24.0 | 120-126 | 17.1 | 88-39 | 7.9/15.7 | 7.8 | 0.46 | 127-129 | 7.0 | 39-45 | 15.7/15.7 | 0.0 | 0.00 |
| D, T22 | Lo | 23.9 | 139-133 | 17.2 | 86-38 | 5.8/14.6 | 8.8 | 0.51 | 132-130 | 6.8 | 38-45 | 15.1/15.7 | 0.6 | 0.09 |
| D, T23 | Hi | 17.5 | 163-172 | 17.5 | 92-39 | 7.2/16.0 | 8.8 | 0.50 | - | - | - | - | - | - |
| D, T23 | Lo | 17.8 | 186-177 | 17.8 | 94-38 | 5.1/14.9 | 9.8 | 0.55 | - | - | - | - | - | - |
| E, T24 | Hi | 18.2 | 191-196 | 11.2 | 60-37 | 12.1/15.6 | 3.5 | 0.31 | 197-200 | 7.1 | 35-33 | 15.8/16.3 | 0.5 | 0.07 |
| E, T24 | Lo | 18.2 | 214-209 | 10.9 | 59-36 | 8.4/14.4 | 6.0 | 0.55 | 208-205 | 7.3 | 34-32 | 14.8-15.7 | 0.9 | 0.12 |

Table S2. Comparison of parts of seabed transects along which temperature is affected or not affected by the tidal front at high and low tide.
