# Supplementary material for: Semidiurnal Temperature Changes Caused by Tidal Front Movements in the Warm Season in Seabed Habitats on the Georges Bank Northern Margin and Their Ecological Implications
Source: PLoS One. 2013 Feb 6;8(2):e55273. doi: 10.1371/journal.pone.0055273 (PMC3566201; doi:10.1371/journal.pone.0055273)
Supplement: Table S3 — Summary of seabed water depths and the frontal effect on temperature change in subareas of study areas A-E. Temperature data was collected at numerous CTD stations located along transects, at time-series stations, and at the starts and ends of video-drift stations. Subareas in each study area are numbered in an off-bank to on-bank direction (Figs. 2,3,4,5,6). Subarea widths are measured normal to the tidal front. Temperature data collected during flood or ebb tides are plotted in Figure S1. The largest temperature changes between high and low tide (ranging from 3.9 to 8.0°C) occurred in subareas that lie in a 11–14 km wide band that extends ∼100 km along the bank margin above 80 m water depth (Fig. 11). Abbreviations: nd – no data; ndef - data not definitive to determine range. (DOC) [file pone.0055273.s003.doc]

Table S3. Summary of seabed water depths and the frontal effect on temperature change in subareas of study areas A-E.

|  |  |  |  |  | Seabed temperature, ^o^C | | | | | |  |
| --- | --- | --- | --- | --- | --- | --- | --- | --- | --- | --- | --- |
|  |  |  | Depth, m | | Subarea | | Flood tide | | Ebb tide | | Date |
| Area, sub | Width, km | CTD sta | Min/Max | Range | Min/Max | Range | Min/Max | Range | Min/Max | Range | Aug. 2009 |
| A,1 | 5.5 | 74 | 52/87 | 35 | 5.7/12.2 | 6.5 | 5.7/10.7 | 5.0 | 5.9/12.2 | 6.3 | 5,6,7,8,10 |
| A,2 | 2.7 | 28 | 49/56 | 7 | 6.7/13.6 | 6.9 | 6.7/11.3 | 4.6 | 7.0/13.6 | 6.6 | 5,6,7,8 |
| A,3 | 3.0 | 33 | 46/58 | 12 | 7.9/13.9 | 6.0 | 7.9/13.9 | 6.0 | 7.9/13.6 | 5.7 | 6,7,8,9,10 |
| A,4 | 2.7 | 11 | 48/56 | 8 | 9.8/13.8 | 4.0 | 11.2/13.8 | 2.6 | 9.8/13.5 | 3.7 | 6,8,9 |
| A,5 | 2.7 | 6 | 53/56 | 3 | 12.4/13.8 | 1.4 | nd | nd | 12.4/13.6 | 1.2 | 6,8,9 |
| A,6 | 7.3 | 6 | 52/56 | 4 | 13.4/14.2 | 0.8 | nd | nd | 13.4/14.2 | 0.8 | 6,8 |
| A,7 | 5.2 | 6 | 51/59 | 8 | 14.0/14.2 | 0.2 | nd | nd | 14.0/14.2 | 0.2 | 4,6 |
| B,1 | 3.1 | 5 | 62/82 | 20 | 6.1/10.0 | 3.9 | 6.1/10.0 | 3.9 | 9.5/9.5 | ndef | 5,9 |
| B,2 | 3.0 | 8 | 50/54 | 4 | 6.9/13.2 | 6.3 | 6.9/13.2 | 6.3 | 13.2/13.2 | ndef | 5,8,9 |
| B,3 | 2.6 | 9 | 51/52 | 1 | 6.7/13.6 | 6.9 | 7.3/13.6 | 6.3 | 6.7/12.4 | 5.7 | 5,8 |
| B,4 | 3.0 | 20 | 46/55 | 9 | 8.7/14.6 | 5.9 | 8.7/14.6 | 5.9 | 9.9/14.2 | 4.3 | 5,8,10 |
| B,5 | 4.1 | 37 | 46/55 | 9 | 12.3/14.7 | 2.4 | 13.3/14.7 | 1.4 | 12.3/14.4 | 1.1 | 4,5,8,10 |
| B,6 | 6.2 | 20 | 48/58 | 10 | 13.5/14.3 | 0.8 | 14.0/14.0 | 0.0 | 13.5/14.3 | 0.8 | 4,5,8 |
| B,7 | 6.4 | 8 | 47/51 | 4 | 14.1/14.3 | 0.2 | nd | nd | 14.1/14.3 | 0.2 | 4,5 |
| C,1 | 3.1 | 4 | 64/81 | 17 | 5.5/11.2 | 5.7 | 7.2/11.2 | 4.0 | 5.6/5.6 | ndef | 9,11 |
| C,2 | 2.7 | 6 | 48/51 | 3 | 7.4/14.2 | 6.8 | 7.4/14.2 | 6.8 | nd | nd | 9,11 |
| C,3 | 2.8 | 21 | 43/47 | 4 | 9.1/14.3 | 5.2 | 9.1/14.3 | 5.2 | 10.0/14.3 | 4.3 | 9,11 |
| C,4 | 2.9 | 11 | 43/46 | 3 | 10.4/14.4 | 4.0 | 10.9/14.0 | 3.1 | 10.4/14.4 | 4.0 | 9,11 |
| C,5 | 2.6 | 6 | 46/50 | 4 | 12.8/14.4 | 1.6 | 14.0/14.0 | ndef | 12.8/14.4 | 1.6 | 9,11 |
| C,6 | 2.8 | 4 | 50/53 | 3 | 13.1/14.7 | 1.6 | 14.2/14.2 | ndef | 13.1/14.7 | 1.6 | 9,11 |
| C,7 | 6.3 | 11 | 46/67 | 11 | 13.8/15.0 | 1.2 | 14.3/14.4 | ndef | 13.8/15.0 | 1.2 | 9,10,11 |
| C,8 | 9.8 | 7 | 54/59 | 5 | 14.8/15.1 | 0.3 | nd | nd | 14.8/15.1 | 0.3 | 9,10 |
| D,1 | 2.2 | 4 | 86/94 | 8 | 5.1/7.9 | 2.8 | 5.1/7.9 | 2.8 | nd | nd | 10,12 |
| D,2 | 2.4 | 8 | 60/76 | 16 | 5.3/13.2 | 7.9 | 5.3/13.2 | 7.9 | nd | nd | 10,12 |
| D,3 | 3.0 | 10 | 42/59 | 17 | 5.8/12.5 | 6.7 | 5.8/12.5 | 6.7 | nd | nd | 10,12 |
| D,4 | 3.0 | 14 | 40/47 | 7 | 7.1/15.1 | 8.0 | 8.9/14.8 | 5.9 | 7.1/15.1 | 8.0 | 10,12 |
| D,5 | 2.8 | 4 | 37/42 | 5 | 11.3/15.7 | 4.4 | 15.3/15.3 | ndef | 11.3/15.7 | 4.4 | 10,12 |
| D,6 | 2.7 | 4 | 37/39 | 2 | 13.8/15.7 | 1.9 | nd | nd | 13.8/15.7 | 1.9 | 10,12 |
| D,7 | 2.6 | 6 | 38/39 | 1 | 14.3/16.0 | 1.7 | nd | nd | 14.3/16.0 | 1.7 | 10,12 |
| D,8 | 7.3 | 6 | 37/45 | 8 | 15.1/15.7 | 0.6 | nd | nd | 15.1/15.7 | 0.6 | 10,12 |
| E,1 | 0.5 | 5 | 59/60 | 1 | 8.4/12.1 | 3.7 | 8.4/12.1 | 3.7 | nd | nd | 13 |
| E,2 | 3.3 | 4 | 40/47 | 7 | 11.1/12.9 | 1.8 | 11.1/12.9 | 1.8 | nd | nd | 12,13 |
| E,3 | 2.9 | 6 | 37/41 | 4 | 12.1/15.3 | 3.2 | 12.1/15.3 | 3.2 | nd | nd | 12,13 |
| E,4 | 4.4 | 34 | 27/38 | 11 | 13.4/15.7 | 2.3 | 13.7/15.7 | 2.0 | 13.4/15.6 | 2.2 | 12,13 |
| E,5 | 2.6 | 14 | 30/35 | 5 | 14.8/16.3 | 1.5 | 15.5/15.7 | ndef | 14.8/16.3 | 1.5 | 12,13 |
| E,6 | 4.2 | 4 | 32/34 | 2 | 15.5/16.4 | 0.9 | nd | nd | 15.5/16.4 | 0.9 | 13 |
